# Micronutrient Status in Adult Crohn’s Disease during Clinical Remission: A Systematic Review

**DOI:** 10.3390/nu15224777

**Published:** 2023-11-14

**Authors:** Martin McDonnell, Stephanie Sartain, Catherine Westoby, Vasiliki Katarachia, Stephen A. Wootton, J. R. Fraser Cummings

**Affiliations:** 1 Human Health and Development, Faculty of Medicine, University of Southampton, Southampton SO16 6YD, UKvasiliki.katarachia@uhs.nhs.uk (V.K.); s.a.wootton@soton.ac.uk (S.A.W.); 2 NIHR Biomedical Research Center, University Hospital Southampton, Southampton SO16 6YD, UK; 3 Department of Dietetics, University Hospital Southampton, Southampton SO16 6YD, UK; 4 Clinical and Experimental Sciences, Faculty of Medicine, University of Southampton, Southampton SO16 6YD, UK

**Keywords:** Crohn’s disease, vitamin, micronutrients, trace elements, minerals, inflammatory bowel diseases, micronutrient deficiency, micronutrient insufficiency, review

## Abstract

Adults with Crohn’s disease (CD) may be at risk of micronutrient insufficiency in clinical remission through restrictive eating, malabsorption, abnormal losses or inflammation. This systematic review synthesises the literature on micronutrient insufficiency in CD in clinical remission in terms of the prevalence of low circulating micronutrient concentrations and as a comparison against a healthy control (HC). Studies were included if the population was predominantly in remission. A total of 42 studies met the inclusion criteria; 12 were rated as low quality, leaving 30 studies covering 21 micronutrients of medium/high quality that were included in the synthesis. Vitamins D and B12 were the most frequently reported nutrients (8 and 11); there were few eligible studies for the remaining micronutrients. The prevalence studies were consistent in reporting individuals with low Vitamins A, B6, B12 and C, β-carotene, D, Magnesium, Selenium and Zinc. The comparator studies were inconsistent in finding differences with CD populations; Vitamin D, the most reported nutrient, was only lower than the HC in one-quarter of the studies. Adult CD populations are likely to contain individuals with low levels of one or more micronutrients, with the most substantial evidence for Vitamins D and B12. The studies on other micronutrients are of insufficient number, standardisation and quality to inform practice.

## 1. Introduction

Undernutrition is a recognised feature of adult Crohn’s disease (CD), both at the time of diagnosis and during subsequent flare-ups when overt nutritional inadequacy is manifested through weight loss [1]. Following the induction of disease remission, dietary restrictions and food avoidance to control CD and its symptoms may continue to alter nutrient and energy intake [2]. Restrictive eating, both clinician and patient-imposed, a loss of absorptive capacity through scarring and resections, increased losses, and an altered metabolic demand from chronic inflammation will put the individual with CD at additional risk of nutritional insufficiency throughout the disease course [3,4,5,6].

Micronutrients are substances that provide structural components and cofactors or substrates for the essential reactions of life. They generally cannot be manufactured by the body and must be consumed in the diet, manufactured by host microbiota, absorbed, and made available to the cells and tissues of the body. CD, its treatments, and sequelae impact these processes and impair the ability of an individual to match their requirements for one or more micronutrients. An impaired micronutrient status may adversely affect wound healing, functional capacity and body structure and put them at increased risk of cardiovascular disease [7,8,9,10].

While international consensus guidelines exist for clinicians on diagnosing and managing iron deficiency anaemia in IBD, there are no guidelines around either the monitoring or treatment of other micronutrients in individuals with CD [11]. The European Society for Clinical Nutrition and Metabolism (ESPEN) consensus guidelines for nutritional care of inflammatory bowel disease (IBD) advocate for regular checking for micronutrient deficiencies but do not specify which micronutrients to monitor or how frequently [12].

Current management of CD seeks to induce and maintain remission, avoid disease-related complications and maintain quality of life with medication, surgery or nutritional therapy [13,14]. Alongside this care, individuals with CD require monitoring and if needed, support of nutritional status. Any blood tests need to be targeted towards the correct patients and clinically relevant markers of micronutrient state. The potential for impaired micronutrient status in CD has apparently been recognised, but the literature has not yet been systematically appraised. To better inform clinical decision-making, there is a need to bring together and appraise the relevant literature on micronutrient status in CD remission.

This systematic review examines the available evidence on micronutrient insufficiencies in adults with CD during clinical remission, in terms of the prevalence (vs. laboratory ranges), comparison to health controls (HCs), contributing factors and clinical consequences of poor micronutrient status.

## 2. Materials and Methods

### 2.1. Protocol and Registration

This review was performed according to PRISMA guidelines with support from university library services. The review was not registered on Prospero.

### 2.2. Eligibility Criteria

The review determined the scope and nature of the evidence for micronutrient insufficiency in adults with Crohn’s disease during clinical remission. Using a PICO strategy, we assigned “P” as adult outpatient CD populations, “I” as blood tests to determine micronutrient status, “C” as pre-defined cut-offs or matched healthy controls for deficiency and “O” as the prevalence of micronutrient deficiency (below pre-defined laboratory ranges) or the serum/plasma micronutrient status in comparison with matched controls. Due to there being meta-analyses on iron status in CD, this was not included in the synthesis.

Secondary outcomes were (1) whether any subgroup analysis was performed to identify associations with disease characteristics (e.g., disease activity score, biomarker) or subgroups (e.g., those with bowel resections), (2) whether any outcome measures of micronutrient state were recorded or (3) whether there was any additional particular test of micronutrient status or stores.

### 2.3. Exclusion Criteria

The following exclusion criteria were used: studies reporting mixed IBD cohorts (without separate reporting of CD subjects), studies that did not report the number or percentage of subjects with deficiency or had no healthy control comparison, or those studies with no recorded description of the clinical disease activity score of the CD cohort.

If results for subjects in clinical remission were not reported separately, studies were excluded if ≥50% had clinically active disease (e.g., CDAI ≥ 150) or where the mean or median disease activity score was above the threshold that defines active disease (e.g., CDAI ≥ 150).

### 2.4. Search Strategy

The following databases were searched in June 2021: COCHRANE, OVID MEDLINE, OVID EMBASE, EBSCO CIHAHL and WEB OF SCIENCE. Additional studies were identified using the references of relevant articles. Searches were restricted to English language articles and no time restriction was placed on articles. Each of these detailed search strategies is shown in Supplementary S1: Search Technique.

### 2.5. Study Selection

Results were saved as a dedicated Endnote library and duplicates were then removed. Titles and abstracts were scanned to select full texts. The extracted full texts were then independently checked by the researchers MM and SAW against exclusion criteria. The reviewer(s) then recorded whether the study met inclusion criteria in the spreadsheet of extracted studies and the results compared. Discrepancies were resolved by re-review and discussion with the final selection of full texts completed in January 2022.

### 2.6. Data Extraction

The following data were extracted into a data table; study first author, year of publication, study size, whether the study compared to reference ranges or HC and micronutrient(s) of the study, and the micronutrient(s) reported in that study (see Table 1). For each micronutrient, a table compiling the studies fulfilling the review inclusion criteria for that nutrient was prepared (see Supplementary S3: Data extracted from eligible studies). In the table for each nutrient, additional information was also recorded. For studies comparing observed values against reference ranges: the percentage of CD subjects with low blood concentration and the cut-off used to define this. For HC studies: the micronutrient concentration in the CD and HC groups, and statistical comparisons between groups. The units of measurement were not converted from those reported initially to avoid altering data as reported. Whether the study concluded that the findings support the view that micronutrient status was impaired among subjects with CD in remission was recorded. Data extracted for secondary outcomes included any correlation of micronutrient level with other disease markers, subgroups of patients or disease outcomes in CD subjects, and whether any particular tests were performed.

### 2.7. Quality/Risk of Bias

Quality was assessed against a checklist based on the principles for observational studies and also considered the potential confounders of observational studies and those specific to assessing micronutrient status in CD [15]. This checklist aimed to systematically capture whether modifiers of micronutrient status were documented and whether the population would be representative of a current cohort. The review-specific confounders added to this checklist included the adequate reporting of subjects’ inflammatory status (as it can alter micronutrient blood tests), previous resections, and micronutrient supplement usage, whether blood tests were prospective, the clarity of the data, how remission was defined, and the percentage of subjects in remission. The components of the study quality checklist are listed in Supplementary S2.

The results of the quality checklist for each study were used to classify the studies as low, medium or high quality in the context of the question addressed in this review. Studies were judged to be low quality if the data were not reported (e.g., only on a graph) or if the confounding variables (resections, inflammatory state, supplement usage or recruitment) were poorly described. Medium-quality studies had clear data but only partial reporting of study subjects’ confounding variables. High-quality studies had clear data and a thorough description of study subjects and confounding variables. Only those of medium or high quality were used to answer the review objectives. The study quality results for each category and overall study classification are displayed in Table 2: Study quality.

The year of the study and whether the cohort contained individuals who were not in clinical disease remission, as well as recognised sources of heterogeneity relevant to the study outcomes, were included in the extracted data tables and referred to in the summary statements for each micronutrient.

### 2.8. Data Synthesis

The extracted data from medium or high-quality studies were then summarised for each micronutrient. Statements were made for each nutrient regarding (1) the range of estimated prevalence of CD individuals below the laboratory reference range, (2) the comparison of CD cohorts to the HC, (3) the cohort characteristics and (4) any exploratory correlations of micronutrient vs. outcome or subgroups. The heterogeneous nature of the studies and the small number of studies for most of the micronutrients precluded any meta-analysis, sensitivity analyses or methods to determine the certainty of the results.

## 3. Results

**Figure 1:**
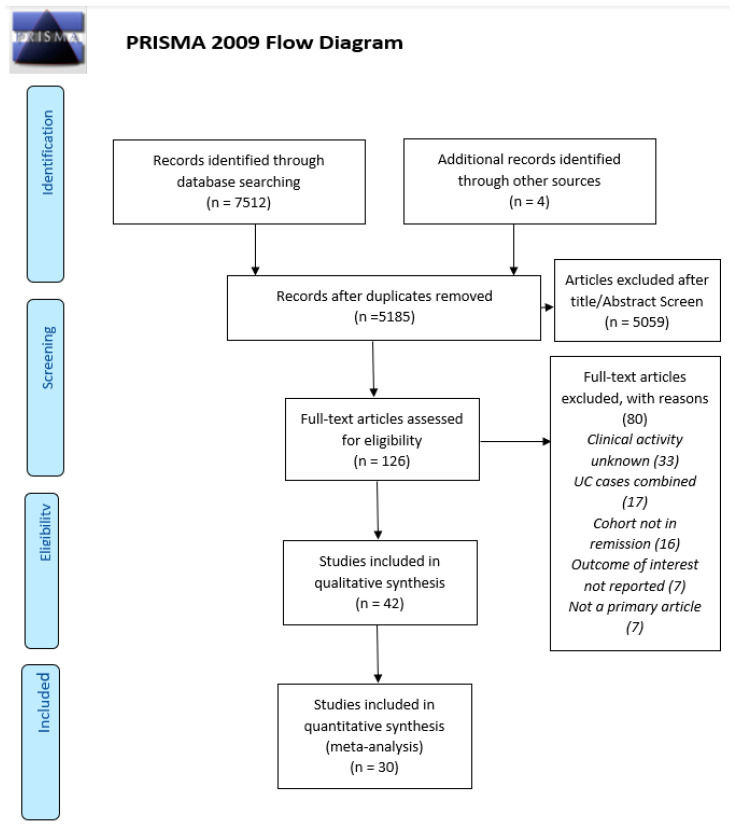
The preferred reporting items for systematic reviews and meta-analyses (PRISMA) flow chart for the selection of studies.

The initial search returned 7512 studies of which 2327 were duplicates. The remaining 5185 titles and abstracts were screened and 5059 were excluded; 126 remaining articles were retrieved for full-text review, of which 85 were excluded (see Figure 1 for reasons). A total of 42 studies met the inclusion criteria for the review, including information on 20 micronutrients (see Table 1).

Less than a third of the studies (13/42) reported micronutrient status in CD remission, either as an inclusion criterion from the study or as a separately reported group. Of these 13 studies, 3 were of low quality and excluded from the data synthesis. Studies of CD subjects with variable clinical activity (which required more than 50% of the cohort to be in remission and the median or mean disease activity score of the cohort was below that used to define remission (e.g., CDAI < 150 or HBI < 5)) made up the remaining 29 studies. The CRP of the study cohort was reported in 19/42 studies.

Of the 42 studies that met the inclusion criteria, 22 reported the percentage of a CD population below a laboratory reference range, 11 compared the micronutrient concentration(s) against a group of matched healthy controls (HCs), and 9 reported both.

The eligible studies reported between 1 and 14 micronutrients, most tended to report on 1 or 2 nutrients (15 were single nutrient studies). There were just five studies that offered a broader micronutrient panel (more than five micronutrients), and these tended to be in small populations, between 23 and 64 subjects [16,17,18,19,20]. One of these broader micronutrient panel studies was from the era of biological therapies [20]. Studies tended to be small with just 11 of the 42 eligible studies reporting on more than 100 subjects, with the larger cohorts being single nutrient studies.

Following the quality assessment, 12 of the 42 selected studies were judged to be of low quality principally due to a lack of clarity in reporting the micronutrient data; these studies were not included in the synthesis.

Sixteen of the thirty medium or high-quality studies that were included in the data synthesis were published after 2010; those published before this time were considered as ‘pre-biologic’.

A list of the eligible studies, the number of CD subjects in each, the type of study, and the micronutrients included in these studies are described in Table 1. The quality of these studies is summarised in Table 2. The data extracted for each micronutrient are recorded in evidence tables in Supplementary S2, and these tables are summarised by nutrient below.

**Table 1:** Summary of studies fulfilling the review inclusion criteria. (“X” denotes that the publication contains information on the Vitamin or mineral of the column, or the type of study described in the column).

					Micronutrient																			
					Liposoluble Vitamins					Hydrosoluble Vitamins								Minerals						
Author	Year	n CD	% Low in CD	CD vs. HC	A	βc	D	E	K	B1	B2	B3	B6	B9	B12	HcY	C	Ca	Cu	Mn	Mg	PO4	Se	Zn
Schoelmerich [21]	1985	54	X	X	X																			X
Imes [22]	1986	137	X														X							
Imes [23]	1987	137	X											X	X									
Geerling [16]	1998	32	X	X	X	X		X		X				X	X		X		X		X		X	X
Geerling [17]	1999	62		X	X	X		X									X		X				X	X
Genser [24]	1999	24		X	X	X		X																
Geerling [18]	2000	23		X	X	X		X		X				X	X		X		X		X		X	X
Koutroubakis [25]	2000	55		X										X	X	X								
D’Odorico [26]	2001	37		X	X	X		X																
Schoon [27]	2001	32		X					X															
Wendland [28]	2001	37		X	X	X		X									X						X	
Duggan [29]	2004	44		X			X		X															
Tajika [30]	2004	33	X	X			X											X			X	X		
McCarthy [31]	2005	44	X	X			X																	
Filippi [19]	2006	54	X	X		X	X	X		X		X	X	X	X		X	X			X	X		X
Gilman [32]	2006	58	X				X																	
Roblin [33]	2007	92	X											X	X	X								
Vagianos [34]	2007	84	X		X	X	X						X	X	X								X	X
Valentini [35]	2008	91	X	X										X	X						X		X	X
Kallel [36]	2011	89	X	X										X	X	X								
Nakajima [37]	2011	47		X		X		X																
Nic Suibhne [38]	2013	81	X	X			X																	
Vagianos [39]	2012	70	X										X	X	X	X								
Bermejo [40]	2013	180	X											X	X									
Garg [41]	2013	40	X	X			X																	
Grunbaum [42]	2013	34	X	X			X																	
Jorgensen [43]	2013	182		X			X																	
Kini [44]	2014	32	X				X																	
Lupu [45]	2015	115	X											X	X									
Soares-Mota [46]	2015	38	X		X																			
Ward [47]	2015	381	X												X									
Basson [48]	2016	44	X				X																	
Battat [49]	2017	66	X												X									
Frigstad [50]	2017	230	X				X																	
Caviezel [51]	2018	99	X				X																	
Branco [52]	2019	106	X				X																	
De Castro [53]	2019	31	X											X	X						X			X
Marcil [54]	2019	274	X											X	X									
Olmedo-Martin [55]	2019	150	X				X																	
Zhao [56]	2019	21	X				X																	
Domislovic [57]	2020	123	X				X																	
MacMaster [20]	2021	59	X		X		X		X	X	X		X	X	X		X		X	X	X		X	X

**Table 2:** Study quality/risk of bias. Assessment against criteria set out in Supplementary S2 study quality checklist as per the description in Section 2.7. Studies of Medium/High quality were included in estimates of prevalence and HC comparison statements. (Abbreviations: “?” denotes that this was unclear in the publication, “n/a” denotes moon applicable to this study as no separate reporting of remission, Crp—C-reactive protein, resect—prior resections, supp—supplement use, Prosp—was the study prospective? sep rem—separate reporting of remission, % rem desc—Percentage of subjects in remission described).

			Reported in CD			Representative of Current IBD Cohorts								
Study	Year	Primary	CRP	Resect	Supp	CRP	Resect	Supp	Recruitment	Prosp	Clear Data	Sep Rem	% Rem Desc	Overall Quality
Schoelmerich [21]	1985	Yes	No	Yes	Yes	?	High	Yes	Yes	Yes	Yes	Yes	n/a	Medium
Imes [22]	1986	No	No	Yes	Yes	?	Yes	Yes	?	Yes	Yes	No	Yes	Medium
Imes [23]	1987	No	No	Yes	Yes	?	Yes	Yes	?	Yes	No	No	Yes	Low
Geerling [16]	1998	Yes	Yes	Yes	Yes	Yes	High	Yes	Yes	Yes	No	No	Yes	Medium
Geerling [17]	1999	No	Yes	Yes	No	Yes	High	?	Yes	Yes	No	Yes	n/a	Medium
Genser [24]	1999	Yes	Yes	Yes	?	High	High	?	?	Yes	Yes	No	Yes	Medium
Geerling [18]	2000	No	Yes	Yes	?	High	Yes	?	Yes	Yes	Yes	Yes	n/a	High
Koutroubakis [25]	2000	Yes	No	No	No	?	?	?	?	Yes	No	No	Yes	Low
Wendland [28]	2001	Yes	No	Yes	Yes	?	Yes	Excluded	?	Yes	Yes	Yes	Yes	Medium
D’Odorico [26]	2001	Yes	No	No	No	?	?	?	?	?	Yes	Yes	Yes	Low
Schoon [27]	2001	No	No	Yes	Yes	?	High	Yes	Unclear	Yes	Yes	Yes	n/a	Medium
Duggan [29]	2004	No	No	Yes	Yes	?	Yes	Yes	Unclear	Yes	No	Yes	n/a	Low
Tajika [30]	2004	Yes	Yes	Yes	Yes	Yes	High	Excluded	No	Yes	Yes	No	Yes	High
McCarthy [31]	2005	Yes	Yes	Yes	Yes	Yes	Yes	Excluded	Yes	Yes	Yes	Yes	n/a	High
Filippi [19]	2006	Yes	Yes	Yes	No	Yes	No	?	Yes	Yes	No	Yes	n/a	Low
Gilman [32]	2006	No	Yes	Yes	Yes	Yes	High	Yes	Yes	Yes	Yes	No	Yes	High
Roblin [33]	2007	No	No	No	Yes	?	?	Excluded	Yes	Yes	Yes	No	Yes	Medium
Valentini [35]	2008	Yes	Yes	Yes	Yes	Yes	Yes	Yes	Yes	Yes	Yes	Yes	n/a	High
Vagianos [34]	2007	Yes	No	Yes	Yes	?	Yes	Yes	Yes	Yes	Yes	No	Yes	Medium
Kallel [36]	2011	Yes	Yes	Yes	Yes	Yes		Excluded	?	Yes	Yes	No	Yes	High
Nakajima [37]	2011	No	No	No	Yes	?	?	Yes	Yes	Yes	No	No	Yes	Low
Nic Suibhne [38]	2012	Yes	Yes	Yes	Yes	Yes	Yes	Yes	Yes	Yes	Yes	No	Yes	High
Vagianos [39]	2012	Yes	Yes	Yes	Yes	Yes	High	Yes	Yes	Yes	Yes	No	Yes	High
Bermejo [40]	2013	Yes	No	Yes	Yes	?	Yes	Excluded	Yes	Yes	Yes	No	Yes	Medium
Garg [41]	2013	No	Yes	Yes	Yes	Yes	Yes	Yes	Yes	Yes	Yes	No	Yes	Medium
Grunbaum [42]	2013	No	No	Yes	Yes	?	Yes	Yes	Yes	Yes	No	No	Yes	Low
Kini [44]	2013	No	No	No	Yes	?	?	Yes	Yes	Yes	No	Yes	Yes	Low
Lupu [45]	2014	No	No	Yes	Yes	?	Yes	No	Yes	Yes	Yes	No	Yes	Medium
Soares-Mota [46]	2015	Yes	No	Yes	No	?	Yes	?	Yes	Yes	No	No	No	Low
Ward [47]	2015	Yes	No	Yes	No	?	Yes	?	Yes	Yes	Yes	No	Yes	Medium
Basson [48]	2015	Yes	Yes	Yes	Yes	Yes	Yes	Excluded	Yes	No	Yes	Yes	n/a	High
Battat [49]	2016	Yes	No	Yes	Yes	?	No	Yes	High	Yes	Yes	Yes	n/a	Medium
Frigstad [50]	2017	Yes	Yes	Yes	No	Yes	Yes	?	Yes	Yes	Yes	No	Yes	Medium
Caviezel [51]	2017	Yes	Yes	Yes	Yes	Yes	High	Yes	Yes	Yes	Yes	No	Yes	High
Branco [52]	2018	Yes	Yes	No	Yes	Yes	?	Yes	Yes	Yes	Yes	No	Yes	Medium
De Castro [53]	2019	Yes	Yes	Yes	Yes	Yes	Yes	Excluded	Yes	Yes	Yes	No	Yes	High
Marcil [54]	2019	Yes	Yes	Yes	No	Yes	High	?	Yes	Yes	Yes	Yes	n/a	Medium
Olmedo-Martin [55]	2019	No	Yes	No	No	High	?	?	Low	Yes	Yes	No	Yes	Low
Zhao [56]	2019	Yes	Yes	Yes	Yes	Yes	Yes	Yes	Yes	Yes	Yes	Yes	n/a	High
Domislovic [57]	2019	No	No	No	No	?	?	?	?	Y	No	Yes	n/a	Low
MacMaster [20]	2020	Yes	No	Yes	Yes	?	Yes	Yes	Yes	Yes	No	No	Yes	Low

### 3.1. Micronutrient Status in CD by Nutrient as Prevalence Compared against Reference Ranges or Compared with HC

#### 3.1.1. Vitamin A (10 Studies)

Four studies, all medium quality, reported the percentage of CD subjects with a serum Vitamin A (retinol) level below the laboratory reference range [16,34,46,48]. The studies reported against different reference ranges, finding 28% (<0.9 µmol/L), 1% (<360 µg/L), 29% (<1.05 µmol/L) and 2% (<1.0 µmol/L) below range. One of these also used a retinol dose response test (an increase of 20% in serum levels following a dose) to assess the adequacy of liver stores, increasing the estimated prevalence of deficiency from 29% to 37% [46].

Seven studies, of which six were of medium or high quality, compared Vitamin A concentrations among CD cohorts that contained both remission and active disease against matched HC [16,17,18,21,24,26,28]. Excluding those low-quality studies, two identified a statistically significant lower Vitamin A among CD subjects in comparison with the HC and three studies reported no difference. One study compared the results of those CD subjects (with CDAI < 150) with and without laboratory changes separately and found only the group with laboratory changes to be lower than the HC [21].

Attempts to determine whether a poor dietary intake could explain the low serum retinol concentration found no association [34]. Dietary Vitamin A was assessed in three other studies, with no difference in intake between those with CD and HC [16,17,28].

Two studies explored the relationship of Vitamin A concentration with clinical disease score; both found no activity.

Subgroup analyses in two studies identified low BMI as predictive of lower Vitamin A [26,46]. Two studies reported a negative correlation between serum Vitamin A and radical trapping antioxidant potential and orosomucoid concentration.

The extent to which Vitamin A status is impaired in CD remission remains uncertain both for prevalence and comparator studies.

#### 3.1.2. β-Carotene (8 Studies)

Three studies reported the prevalence of serum β-carotene concentrations below laboratory reference ranges, of which one was of low quality [19]. The other two studies were mixed CD cohorts of variable disease activity, each with a different cut-off to define deficiency (0.4 µmol/L and 1.0 µmol/L). One of these studies reported a deficiency in all the CD cohorts, the other in 29% [16,34].

Seven studies compared serum β-carotene concentrations between CD subjects and the HC [16,17,18,19,24,26,28]. Five of these studies were from the pre-biological era of treatment and four had variable disease activity cohorts. Four of five studies identified significantly lower β-carotene levels among CD subjects, one of which was the study that had clinical remission as an inclusion criterion [16,17,24,28].

Exploratory analyses of correlates of β-carotene levels demonstrated a relationship with lower estimated Vitamin A intake, a negative correlation with disease activity index, and lower levels among those with active disease [17,26,34].

The literature supports there being individuals with serum β-carotene levels below laboratory reference ranges among populations with CD. A greater number of studies compare β-carotene concentrations between CD subjects and the HC, which generally support levels being lower in CD than in the general population.

#### 3.1.3. Vitamin D, Specifically, 25-OH Vitamin D (20 Studies)

Sixteen studies reported the prevalence of low serum concentrations (defined as deficiency or insufficiency typically defined as <50 nmol/L) of 25-OH Vitamin D (25-OH D) in CD cohorts, four compared with the HC and five reported on both [19,20,31,32,34,38,41,42,44,48,50,51,52,55,56,57].

Thirteen of the prevalence studies that reported the proportion of CD subjects with a low 25-OH D, were of medium or high quality. The reported percentage of low Vitamin D in these populations ranged from 14% to 76% [20,30,31,32,34,38,41,44,48,50,51,52,55]. Four of these had either clinical remission as an inclusion criterion or separately reported those in remission [20,31,48,55]. One of these remission-only studies had the lowest percentage of CD subjects with a low 25-OH D, while the highest was reported among a cohort of variable disease activity (median CDAI 106) in the sample taken during winter months [44,48]. Four studies with HC comparators were of medium or high quality, including one of the studies with separate reporting of those in CD remission [30,31,38,41]. Of these, just one study reported a significantly lower 25-OH D concentration (29%) in those with CD than the HC [31].

Exploratory analyses identified lower 25-OH D levels in those with higher clinical disease scores and higher faecal calprotectin. One study reported a correlation between dietary Vitamin D intake and serum 25-OH D concentrations [34]. 25-OH D was consistently lower in winter months and related to supplement use, disease duration and subtype [32,38,48,55].

Vitamin D deficiency, as marked by 25-OH D concentrations below 50 nmol/L, is frequently observed in adults with CD in clinical remission. Fewer studies compare 25-OH D concentrations between CD and an HC, but they do not support deficiency being more of an issue among those with CD in remission than the rest of the population. The large variability in reported prevalence may reflect seasonality, country of study, supplement use and disease activity.

#### 3.1.4. Vitamin E (7 Studies)

Three studies reported on the percentage of CD subjects with a Vitamin E below the reference range, one of which was of low quality [16,19,20]. The remaining two studies used different cut-offs, had different inclusion criteria, and were from different eras of treatment. The first was in a cohort of variable disease activity in the pre-biological era and reported 45% below the reference range (<14 µmol/L). The other study had CDAI < 150 as an inclusion criterion, corrected for serum cholesterol, defining a low Vitamin E level as <3.5 µmol/mmol cholesterol and reported 2% of cases below the reference range.

Seven studies compared levels between CD and the HC, two were of low quality [16,17,18,19,24,26,28]. Of the five studies of either medium or high quality, three found significantly lower Vitamin E levels among CD subjects, and two no difference.

Serum Vitamin E concentrations were correlated to serum cholesterol in two studies [16,18]. Two studies found no correlation between Vitamin E concentrations and clinical disease activity makers [26,28].

The likely prevalence of a reduced serum Vitamin E level among subjects with CD remission cannot be determined from the limited literature. The HC comparator studies are more numerous, yet inconsistent in their findings. Some HC comparator studies suggest lower Vitamin E levels among those with CD than in the general population whilst others do not. Vitamin E levels appear to be correlated to serum cholesterol levels but not with markers of CD activity. Variability in correcting for serum cholesterol between studies is likely to be a factor in the inconsistency between different HC comparator studies and prevalence studies.

#### 3.1.5. Vitamin K (4 Studies)

One prevalence study found that 2% of the cohort had Vitamin K levels below a laboratory reference range (corrected for serum triglycerides) [20]. Three studies compared Vitamin K status among subjects with CD and the HC. Of these, one study [27], was of medium quality and two were of low quality [29,37]. The study of medium quality, in which all subjects were in clinical remission, reported both serum Vitamin K and the concentration of Undercarboxylated Osteocalcin as a marker of Vitamin K status [27]. Vitamin K concentrations were lower and undercarboxylated osteocalcin was higher, among CD cases. This maker was inverse to bone mineral density (BMD) in the lumbar spine.

Taken together, the literature on Vitamin K status in CD is limited, varied in the definitions for deficiency used, and cannot currently establish the prevalence of a low serum Vitamin K among those with CD in remission, nor is it adequate to establish whether levels are lower in comparison with the population in general. One study of moderate applicability and medium quality suggests that Vitamin K status is more impaired among those with CD than in the general population.

#### 3.1.6. Thiamine (B1) (4 Studies)

Four studies, one of low quality, compared Thiamine concentration among a CD population with either a laboratory reference range or the HC [16,18,19,20]. One study from 2021 with HBI ≤ 4 as an inclusion criterion found no subjects below the reference range of <275 ng/g Hb [20].

The other two studies, which included CD subjects of variable clinical activity, identified no significant difference in Thiamine concentrations compared to the HC [16,18]. One of these studies also found no cases below the reference range of <85 nmol/L [16].

This limited literature finds no evidence of low Thiamine among CD subjects.

#### 3.1.7. Riboflavin (B2) (1 Study)

One prevalence study was found and reported only 1/59 (2%) cases had a Riboflavin concentration below the reference range (<1.0 nmol/g Haemoglobin (Hb)) [20]. No comparator studies were identified that compared Riboflavin levels with that of the HC.

This limited literature is insufficient on the prevalence of reduced Riboflavin levels among CD subjects and, in comparison to, the general population.

#### 3.1.8. Niacin (B3) (1 Study)

One low-quality study reported on both the prevalence of Niacin deficiency in a CD population and compared levels with a matched HC [19].

Taken together, there is insufficient literature to determine the prevalence of reduced Niacin among CD subjects and, in comparison to, the general population.

#### 3.1.9. Pantothenic Acid (B5)

No studies which met the inclusion criteria reported on Pantothenic acid.

#### 3.1.10. Pyridoxic Acid (B6) (4 Studies)

Four studies found the prevalence of B6 concentrations below the lower reference range, one of which was of low quality [19,20,34,39]. The low-quality study was the only one to compare B6 levels with the HC [19].

The three remaining studies reported a lower B6 in 45.7%, 30% and 10%, which they defined as <5 µg/L, <20 nmol/L and <25 pmol/g Hb, respectively. The two studies with higher prevalence were of CD subjects with varying clinical activity and were both assessed as being of medium quality [34,39]. The study with the lowest prevalence (10%) used the B6 concentration per gram of Hb to correct for the acute phase response and had CDAI < 150 as a study inclusion criterion [20].

Both studies that reported a higher prevalence of low B6 also assessed the intake of B6 with food frequency questionnaires and identified a correlation of serum B6 with estimated intake. The other study found B6 concentrations were lower among subjects with stricturing rather than the inflammatory disease phenotype [20].

The limited literature consistently supports a low serum B6 being present in subjects with CD in remission and that dietary intake and serum levels are related. No studies of sufficient quality meet the review inclusion criteria to compare B6 concentrations between CD populations and the HC.

#### 3.1.11. Biotin (B7)

No studies which met the inclusion criteria reported on Biotin.

#### 3.1.12. Folic Acid (B9) (15 Studies)

Twelve studies reported the percentage of CD subjects with either serum or red blood cell Folate below a laboratory reference range, of which five were of low quality [19,23,45,54]. Different cut-offs were used across the seven studies of medium or high quality [20,33,34,35,39,40,53]. Four of these studies, of which two had remission as a study inclusion criterion, reported either none or just one deficient subject in the CD cohort [34,35,39,53]. Two studies of CD subjects with mixed clinical activity, with cut-offs to define deficiency of <3 ng/mL and RBC Folate <210 nmol/L, reported a much higher prevalence (22% and 35%, respectively) [33,40].

Six studies, two of low quality, compared levels against the HC [33,40] and found no difference in Folate levels between mixed CD remission populations and HC [16,18,35,36]. Exploratory and correlation studies identified a correlation with dietary intake in two studies and an inverse correlation with Homocysteine, clinical activity and radiological evidence of active disease [36,40,53].

Taken together, the literature on the prevalence of low Folate is inconsistent regarding how deficiency is defined and whether there are individuals with a low level in the population. The three comparator studies do not support lower Folate concentrations among those with CD than in the general population. The exploratory analyses suggest that low Folate may be in part related to dietary intake and disease activity.

#### 3.1.13. Cobalamin (B12) (18 Studies)

Thirteen studies reported on the prevalence of a low B12 (below a reference range) or other biochemical evidence of an impaired B12 status (such as a Holotranscobalamin (holoTC) below the reference range or methylmalonic acid (MMA) above the reference range) [19,20,23,33,34,35,39,40,45,47,49,53,54]. Nine studies, which were of medium or high quality, identified CD subjects with evidence of impaired B12 status [20,33,34,35,39,40,47,53]. The highest prevalence (33%) was among a group of CD subjects in clinical remission which defined an impaired B12 status as either a holoTC below 2 or a holoTC 25–50 with a paired MMA > 280 [47]. The study with the lowest reported percentage of B12 deficient subjects (4%) defined this as hydroxocobalamin < 197 pg/mL and remission through both radiological and endoscopic criteria [53].

Six studies compared B12 concentrations with the HC, of which two were of low quality [16,18,19,25,35,36]. B12 concentrations were lower among the CD group in two of the four medium or high-quality studies and did not differ in the other studies.

In the exploratory and correlation studies of B12 concentrations, three studies identified ileal resections as a risk factor for a B12 below the reference range [40,47,49]. Two of these studies specified the length of ileal resection associated with this, one 20 cm and the other 30 cm [47,49]. The former study also found lower B12 concentrations among those with terminal ileal inflammation [47]. Two other studies found no relationship between B12 with disease activity. Two studies found a correlation between dietary B12 intake (per 100 kcal diet), and both identified a correlation with serum B12 [34,39]. The relationship of B12 concentration to Homocysteine concentration was reported in three studies of medium or high quality; two found a negative correlation, and the other had no relationship [33,36,39].

The data on impaired B12 status in CD remission are heterogeneous but consistently support evidence of cases with low B12 concentrations in the population. The smaller number of HC studies suggests that B12 levels are likely lower in CD populations than in the general population. The correlation analyses support ileal resections with ileal inflammation being a risk factor for B12 deficiency and dietary intake contributing to serum B12 levels [33,36,39].

#### 3.1.14. Homocysteine (HcY) (3 Studies)

Homocysteine elevation can arise due to inadequacy in one or more of Vitamins B2, B6 and B12 and was therefore reported separately in this systematic review. Three studies reported the prevalence of hyperhomocysteinemia in a CD population. Each included some individuals with clinically active disease and found hyperhomocysteinemia to be present among between 13.7 and 60% [33,36,39].

Two studies compared HcY concentrations between CD populations with the HC, one of low quality and the other of high quality. The high-quality study, in a population of CD subjects of whom some had clinically active disease, found significantly higher HcY levels among CD subjects than the HC [36].

Correlation analyses identified hyperhomocysteinemia as being associated with osteoporosis [33]. Two studies found B12 concentration negatively correlated with HcY concentration [36,39]. One of these studies also used multivariate analysis to identify Folate level (negative), age, active disease and disease duration (all positive) as being associated with serum hyperhomocysteinemia [36].

The limited literature supports hyperhomocysteinemia being present in populations with CD. One well-designed study supports HcY concentrations being more elevated in CDD than in the general population.

#### 3.1.15. Vitamin C (7 Studies)

Four studies, one of low quality, reported the percentage of subjects with Vitamin C below reference ranges [16,19,20,22]. All the higher-quality studies reported cases with low Vitamin C concentrations. The two studies with the highest percentage prevalence were both cohorts of variable disease activity in the pre-biological era; the first reporting a low serum Vitamin C concentration (<30 µmol/L) in 15% and low leucocyte ascorbate (<0.7 µmol/109 cells) in 37%, and the other, a low serum Vitamin C concentration (<11 µmol/L) in 50% of participants [16,22]. The most recent study, which required HBI ≤ 4 for study entry, found a low Vitamin C (<15 µmol/L) in 17% of subjects [20]. Five studies compared Vitamin C levels between the CD population and HC, of which one was of low quality. Of the remaining studies, two found a lower Vitamin C among CD subjects and two studies identified no difference [16,17,18,19,28].

Two studies reported Vitamin C intake among subjects with CD, identifying dietary inadequacies; however, neither reported the relationship between intake and blood concentration [16,22]. Three studies explored the relationship between clinical disease activity score and Vitamin C concentration; two found no relationship, and one had a negative correlation [17,22,28].

The literature consistently suggests that there are likely to be subjects with Vitamin C concentrations below reference ranges in adult CD cohorts. The HC comparator studies are more numerous, but not contemporary and inconsistent in their findings. Dietary inadequacy may be present among CD populations, but its relationship to blood concentrations has not been explored.

#### 3.1.16. Calcium (2 Studies)

Only one study, which was of low quality, reported the prevalence of a low serum Calcium. Two comparator studies, one of low quality, compared Calcium concentration to the HC. The study of high quality failed to demonstrate any difference in Calcium concentration in CD subjects compared to the HC [19,30]. The same study found no relationship between Calcium and 25-OH D concentrations.

Taken together, there is insufficient evidence to estimate the prevalence of a low serum Calcium during CD remission and whether subjects with CD are likely to have lower serum Calcium concentrations than the HC.

#### 3.1.17. Copper (4 Studies)

Two studies, both of medium quality reported on the percentage of CD subjects with a serum Copper level below laboratory reference ranges [16,20]. One, in the pre-biologic era that included subjects of variable disease activity, did not have any subjects with a serum Copper level below the normal range; the other, in a 2021 study for which the inclusion criteria was a CDAI < 150, reported 5% below the reference range. The pre-biologic era study also compared Copper levels with the HC, as did two other studies which were also by the same author; one was of medium quality and one of high quality [16,17,18]. All three studies were in mixed CD populations predominantly in clinical remission and reported no difference in Copper levels vs. HC. One study found Copper levels to be higher among those with clinically active disease [17].

Taken together, the limited literature from the pre-biologic era does not support that there is a difference in Copper concentrations between individuals with CD and the general population. There is inadequate literature to determine whether there are likely to be individuals with low Copper in the population with CD during remission.

#### 3.1.18. Manganese (1 Study)

One study of medium quality reported on the prevalence of a low serum Manganese level in a cohort of individuals, all of whom were in clinical remission, and found no subjects below the laboratory reference range [20]. There were no HC studies.

#### 3.1.19. Magnesium (7 Studies)

Five studies, one of low quality, reported on the percentage of subjects with serum Magnesium below the laboratory reference range [16,19,20,35,53]. Of the four medium or high-quality studies, two were in the pre-biological era; the first reported a low Magnesium in 50% of a cohort of variable clinical activity, and the other was 28.7% in a study with CDAI remission as an inclusion criterion. Of the more recent studies of medium or high quality, one from 2019, which required either radiological or endoscopic evidence of “deep remission”, and the other from 2021, which required a CDAI < 150, reported low Magnesium in 15% and 10%, respectively [20,53]. Five studies, all in the pre-biologic era of which one was of low quality, compared Magnesium levels in a CD population with the HC [16,18,19,30,35]. Two of the four studies of medium or high quality, both in subjects of variable clinical activity, found a lower serum Magnesium level than in the HC [16,30]. The other two studies, one in a mixed disease activity cohort, and the other in a study with CDAI < 150 as an inclusion, found no difference [18,35].

The small body of literature including well-characterised cohorts of adults in CD remission consistently finds that populations with CD in remission contain individuals with a low serum Magnesium. The studies that compare Magnesium concentrations in CD to the HC are inconsistent in showing that the levels among mixed CD populations are lower than in the general population.

#### 3.1.20. Phosphorous (2 Studies)

One study, which was a low-quality study, reported on the prevalence of a low serum Phosphorus concentration [19]. One study, which was of high quality and in a CD population containing both active disease and remission, compared Phosphorus levels with the HC. This study found levels lower among the CD population [30].

#### 3.1.21. Selenium (6 Studies)

Three studies compared Selenium to a reference range [16,20,35]. The two studies in the pre-biological era, one in a cohort of subjects with variable CD clinical activity, and the other in a cohort all in clinical remission, reported low Selenium concentrations (defined as <0.91 µmol/L and <0.59 µmol/L) in 50% and 61.7%, respectively [16,35]. The most recent study from 2021, in which subjects required a CDAI < 150, found 5% of subjects below the serum cut-off (<0.75 µmol/L) [20].

The two pre-biological era prevalence studies also compared concentrations to the HC; the variable clinical activity cohort found that on average, Selenium values were 17% lower among the CD cohort than the HC, and the other study (all CDAI < 150) found Selenium levels were lower among men (vs. HC), but not women. Three other studies, all medium quality, compared Selenium levels in pre-biological CD cohorts with the HC; one found Selenium concentrations were 13% lower among CD than the HC, and the other two studies found no difference [17,18,28].

Two studies found no significant correlation between Selenium concentrations and disease activity [17,28]. One found no relationship between Selenium status and breath markers of redox state [28].

A small body of literature suggests that Selenium deficiency may be prevalent among CD populations during clinical remission. The literature on whether Selenium levels are lower among those with CD than in the rest of the population is inconsistent.

#### 3.1.22. Zinc (9 Studies)

Seven studies, one of low quality reported on the percentage of CD subjects with Zinc concentrations below laboratory reference ranges [16,19,20,21,34,35,53]. Of the six studies of medium or high quality, two in CD cohorts of mixed clinical activity, two in a cohort in CDAI remission, one with separate reporting of CDAI remission subjects the other in subjects with radiological/endoscopic remission all reported subjects with low Zinc levels, ranging from 4.3 to 50% [16,20,21,34,35,53].

Six studies, one of which was of low quality, compared Zinc levels between CD subjects and the HC [16,17,18,19,21,35]. Three studies found Zinc concentrations to be significantly lower among CD subjects than the HC. These studies also compared CD to reference ranges and identified low Zinc concentrations in the CD population; 35% in a mildly active group with low CDAI and abnormal bloods, 12.5% in low CDAI and “normal bloods”, 11% in a cohort of variable clinical activity and 5% lower among a separately reported clinical remission group [16,17,21]. Two other HC studies, one in a cohort with variable clinical activity, and the other in a cohort with CDAI all <150, found no difference in serum Zinc concentrations in CD in comparison with the HC [18,35].

Zinc concentrations were not related to either disease activity or estimated intake in exploratory analyses. Of note, a lower serum Zinc concentration was predictive of a shortened time to clinical relapse in a 12-month prospective cohort study [20].

The limited literature consistently supports there being subjects with serum Zinc concentrations below the laboratory reference range among subjects with CD during remission. The smaller literature body of comparing Zinc levels in subjects with CD is inconsistent.

### 3.2. Summary Statement for Micronutrient Status in CD

Taken together, the available evidence supports the view that there are likely to be individuals with low circulating concentrations of Vitamins A, B6, B9, C, D, E and β-carotene and the minerals Magnesium, Selenium and Zinc in adult CD populations during clinical remission. Of these, the most secure estimated prevalence of individuals at risk of deficiency is most secure for Vitamins D and B12, for which there is a larger body of literature (11 and 8 eligible studies, respectively). For the nutrients Vitamins B2, B3 and B5 and the minerals Calcium, Manganese and Phosphorous, there were no eligible studies identified to estimate the percentage prevalence of low values.

For the studies that compare adult CD remission populations with a matched healthy control (HC), at least half of the eligible studies support the following micronutrients being reduced among CD populations; lower concentrations of Vitamins B12, C and K and β-carotene and the minerals Magnesium and Phosphorous were reported among CD subjects in at least half of the HC comparator studies. For Vitamin B9 (Folate) and Copper, all eligible HC studies found no difference between CD and HC (3 studies and 2 studies, respectively). Fewer than half of the studies of Vitamins A, C, D and E and the minerals Selenium and Zinc found that subjects with CD had lower levels than that of the HC, so the evidence was deemed uncertain. There were no HC comparator studies for Vitamins B2, B3 and B6 and Manganese to assess whether these micronutrients were lower among individuals with CD.

The ranges of reported prevalence of low micronutrient concentrations in comparison to laboratory ranges and against the HC for eligible studies for each micronutrient are summarised in the table below (see Table 3). 

## 4. Discussion

This review identified a body of literature on micronutrient status among subjects with CD, both in comparison with normal reference ranges and against a matched HC. For the majority of the 14 vitamins and 7 minerals and trace elements in the review, there were insufficient studies, both in number and quality, to address the primary research questions. Generalisable conclusions were also challenged by the studies being from populations that were heterogeneous in terms of treatment, disease activity and previous surgery. The data were generally taken from cross-sectional studies of mixed CD populations. The quality of the studies was limited by incomplete reporting of confounders such as recruitment methods, inflammatory markers, previous surgery and supplement consumption in the CD cohorts. The estimated prevalence of low circulating levels of other micronutrients in CD remission and HC comparisons was further limited by differences in the choice of the cut-off and by the number of recent studies of adequate quality.

There appear to be insufficient studies of adequate quality to provide secure estimates of the prevalence of biochemical signs of micronutrient deficiencies. However, there are consistent reports that indicate that some, but not all, patients with CD in clinical remission will have a low circulating concentration of the following micronutrients: Vitamins A, B6, B12 and C and β-carotene and the trace elements Magnesium, Selenium and Zinc.

Of these, only the data for Vitamins D and B12 were from adequate size and quality studies to draw firm conclusions about whether this is likely to represent CD subjects in remission in general and the likely proportion of individuals at risk for deficiency. A widely cited narrative synthesis of micronutrient status in adult CD describes micronutrient deficiencies as “common” in adult CD but is not clear what this means in numerical terms or what the threshold for this term might be [58]. A consistent and clear definition of what is “common” and a wider literature is needed to inform decisions around the scope of blood monitoring needed and justifiable in the IBD clinic.

When the 42 studies were categorised into low, medium, or high, using the study quality checklist, 12 were excluded from the summary statements of each micronutrient. At the expense of removing confounders from the estimates of prevalence or micronutrient status difference between CD and a HC, we may have inadvertently excluded some relevant studies. The review also excluded studies in which the data of individuals with UC were combined in the results, which may have excluded well-designed studies to better understand the micronutrient state in adult CD or IBD in general. One study with combined UC and CD found distinct eating habits and antioxidant vitamin status in both active and remission IBD when compared with the HC [59].

Micronutrients are not consumed, absorbed and metabolised in isolation. None of the studies in the review that report on more than one micronutrient or metabolite provide data that consider this issue. The studies do not report their findings in such a way as to determine whether those individuals with a low concentration of one particular micronutrient were more likely to be low on others.

The second key question of the review was whether the micronutrient status was lower among the CD population in remission than the general population. The data available to answer this were also limited. There were either no eligible studies or only one study for nearly half of the micronutrients. The largest body of data was for Vitamin D; this review found no significant difference between the HC and CD for four of the five eligible studies. A meta-analysis which concluded that levels were lower in CD than the HC may reflect the inclusion of individuals with active disease, which itself is a risk factor for a lower 25-OH D [60,61,62].

The only nutrient with more than one eligible HC study that was consistent in its findings was Folate, with all three studies finding no difference between CD and the HC.

When initially scoping the literature, it was apparent that the number of studies in which the results of subjects in remission were reported separately was limited. Of the 40 eligible studies, just 13 reported the micronutrient status of those in clinical remission separately and only 10 of these were of sufficient quality for the review. This necessitated the inclusion of data from CD cohorts of mixed clinical activity. This was particularly necessary for certain less commonly reported nutrients as studies of micronutrient status tended to focus on specific nutrients. Of the 39 eligible studies, 20 of them included Vitamin D, 13 included B12, and 11 included B9. Aside from Vitamin B12, which is absorbed in the terminal ileum, whether impairment of these three nutrients is particular to Crohn’s disease or is of specific clinical consequence, is not clear.

The year of the study is likely to have been a factor in the nutritional status of the cohorts studied. Landmark CD studies shortly before 2010 demonstrated the efficacy of biological therapy as a disease modifier [63,64]. In countries with adequate resources, this led to the more widespread adoption of biological therapies for moderate to severe Crohn’s This is likely to have an impact on the disease activity of the cohort, prevalence of overt malnutrition, risk of surgery and thus micronutrient status. In the narrative summary for each nutrient, 2010 was therefore used as the cut-off for the “pre-biologic” era.

Where explored and reported in the included studies, micronutrient blood concentrations tended to be negatively correlated with disease activity scores. This was a consistent finding in the studies of Vitamin D, Folate and β-carotene. The degree of impairment of these micronutrients among subjects with CD in remission is therefore likely to have been overestimated by the inclusion of cohorts containing some subjects with active disease. Systemic acute inflammation is negatively correlated with circulating micronutrient concentration and there is an increasing recognition that reduced serum micronutrients in IBD may be inflammation-associated epiphenomena [65,66]. The variable reporting of this between studies is likely to account for the variable prevalence of deficiency seen in the studies included.

There were multiple challenges in drawing generalisable conclusions about micronutrient status in CD remission from the available literature. Eligible studies covered a 30-year period of CD cohorts, a time in which the treatment armamentarium and goals of therapy were transformed by the availability of biological therapies. As such, factors which may in themselves impact micronutrient status such as the degree of inflammation, scarring from previous inflammation and length of resected bowel will differ by era of study. The eligible studies which reported on multiple micronutrients (other than a Vitamin D-focused study) tended to be from the pre-biological era, so the applicability of data on clinically relevant micronutrients such as Thiamine to a current treatment cohort is particularly uncertain.

In the studies reporting on the prevalence of micronutrient deficiency, there was variability between eligible studies for each nutrient in the cut-offs, methods and units used. To inform the identification of subjects at risk of micronutrient deficiency, studies should report SI units and clinically relevant cut-offs, and be consistent in what proportion of deficient cases constitutes a ‘common deficiency’.

The methods and criteria used will have impacted on estimates of prevalence of impaired micronutrient status. Using B12 status as an example, while most studies measured serum total Cobalamin, the study which used measured Holotranscobalamin, likely a more reflective marker of B12 status and MMA when the holoTC was indeterminate as a functional measure of status, suggested 33% of CD subjects had an impaired B12 status, the highest estimate prevalence from the studies included [47,67]. This may mean that inadequacy among the other studies and summary statements was underestimated. Studies of other nutrients also varied in the methods and adjustments used to define deficiency. For example, one group measured B6 per gram of Haemoglobin, to avoid potential confounding for an inflammatory state, while other studies did not do this [20,34,39,68]. The same group also expressed α-tocopherol per mmol of cholesterol, to avoid a previously described confounding of acute inflammation serum levels but was the only eligible study to do so [69]. Such a variability between studies in the definitions and adjustments to identify those at risk of micronutrients is another challenge in synthesising and interpreting the literature.

The units of measurement and statistical approaches used for HC comparisons differed across the studies. Therefore, the data were not deemed suitable for combination meta-analyses. The correlation of each nutrient with disease subtype, disease activity, diet and clinical consequences was not consistently performed across the eligible studies. Some studies’ primary aim was to explore the relationship of one or more of these factors with micronutrient status; for others, it was reported as a secondary outcome or exploratory observation. 

The important clinical question as to whether the deficiencies seen across a range of micronutrients within study populations occur in the same individuals is not answered in the way that the studies are reported. It is not therefore clear whether being low in a specific more commonly checked nutrient or having certain disease features could serve to identify a targeted approach, whereby only certain adults with CD routinely undergo a broader micronutrient profile. The studies identified also had limited exploration of which factors such as diet, previous surgery or inflammation may determine micronutrient status, and apart from bone mineral density, there was no consistent reporting of micronutrients against clinical outcomes.

Nutrition is a key area of interest to patients with IBD, it is therefore important for clinicians to have an evidence base on which to form their advice and monitoring. There is a need to establish which individuals within CD populations are most likely to have micronutrient deficiencies during clinical remission, establish which disease and dietary factors may determine this and explore the consequences of this. Evidence for the therapeutic benefit of dietary manipulation through the removal of potentially pro-inflammatory elements is emerging, yet discussion around what nutrients may likely be dietarily inadequate for individuals with CD and the consequences of those inadequacies needs equal consideration for care [69].

With enhanced biomarker monitoring and effective medical treatments, the treatment paradigm of CD is changing towards goal-directed therapy. IOBD Stride-2 criteria now seek the normalisation of health-related quality of life as a long-term treatment target. There is a need to better understand the extent to which micronutrient status contributes to impairment in quality of life [14].

There is a need for a broad nutritional and phenotypic characterisation of micronutrient status against population norms or healthy controls alongside relevant outcomes in an adult CD remission population. An understanding of the pattern of inadequacy, determinants of nutrient inadequacy, and relationship between different micronutrients to one another and the outcome is needed to establish how to best direct nutritional monitoring and support. Confounders such as age, inflammatory state and previous surgery need to be included, and diet, including supplement usage, also needs to be included in such analyses. With more detailed reporting, the extent of the unmet nutritional needs of the CD population can be better determined and used to better direct care and monitoring towards those subjects in the CD population most likely to derive meaningful benefit. This was a comprehensive and systematic review of a varied literature on micronutrient status in CD remission. Studies may have been missed, and the addition of several eligible papers identified in the references of the initial extraction suggests that some articles may have not been picked up on database searches.

## 5. Conclusions

This systematic review finds that populations of adults with CD during clinical remission are likely to have a range of micronutrient deficiencies. With the exception of Vitamins D and B12, the evidence base to identify which biochemical deficiencies are likely to be present and the prevalence is insecure. The literature comparing micronutrient status in CD remission to a HC is also limited and is therefore unable to predict which nutrients are likely to be lower in CD than the general population There is a need for studies comparing well-characterised cohorts of adult patients with Crohn’s disease in clinical remission cohorts to reference ranges and a HC against clinically relevant outcomes to inform patient care.

## Figures and Tables

**Table 3:** Summary of Prevalence of Insufficiency in CD and comparison to Health Controls for eligible studies.

	Prevalence Studies				Healthy Control (HC) Studies		
Micronutrient	Number of Studies (Total Number of Subjects)	Range of Reported Lower Cut-Offs	Reported Prevalence (%)	Evidence of Deficiency in CD Population	Number of Studies vs. HC (n) Year	Number of Studies in Which CD < HC	Evidence Supports CD < HC
Retinol (Vitamin A)	3 (154)	0.9–1.2 µmol/L	1–29	Yes	5 (178)	2/5	Uncertain
β-carotene	2 (116)	0.4–1 µmol/L	29–100	Yes	5 (178)	4/5	Yes
Vitamin D	11 (968)	25–50 nmol/L	14–76	Yes	4 (198)	1/4	Uncertain
Vitamin E	1 (32)	14 µmol/L	45	Yes	5 (178)	3/5	Uncertain
Vitamin K	None	-	-	Unknown	1 (32)	1/1	Yes
B2/B3/B5	0	-	-	Unknown	0	-	Unknown
Vitamin B6	2 (154)	5 µg/L	30–46	Yes	0	-	Unknown
Vitamin B9	6 (551)	6.8–11.8 nmol/L RBC 210–725 nmol/L	0–35	Yes	4 (238)	0/4	No
Vitamin B12	8 (998)	Low holoTC or high MMA or B12 < 197 pg/ml	4–33	Yes	4 (238)	2/4	Yes
Homocysteine	3 (251)	13–15 µmol/L	14–60	Yes	1 (89)	1/1	Yes
Vitamin C	2 (169)	11–30 µmol/L	15–50	Yes	4 (154)	2/2	Yes
Calcium	0			Unknown	1 (33)	0/1	No
Copper	1 (32)	12 µmol/L	0	Unknown	3 (117)	0/3	No
Manganese	0	-	-	Unknown	0	-	Unknown
Magnesium	3 (157)	0.75 mmol/L	15–50	Yes	4 (182)	2/4	Uncertain
Phosphorous	0			Unknown	1 (33)	1/1	Yes
Selenium	2 (157)	0.59–0.91 µmol/L	50–61	Yes	5 (248)	2/5	Uncertain
Zinc	2 (129)	10–10.3 µmol/L	50	Yes	4 (211)	2/4	Uncertain

## Data Availability

No new data were analyzed in this study. Data sharing is not applicable to this article.

## Supplementary Materials

The following supporting information can be downloaded at: https://www.mdpi.com/article/10.3390/nu15224777/s1, Supplementary S1: Search Technique, Supplementary S2: Study Quality Checklist, Supplementary S3: 21 Data tables on extracted data for micronutrients.
