# Supplementary material for: Micronutrient Status in Adult Crohn’s Disease during Clinical Remission: A Systematic Review
Source: Nutrients. 2023 Nov 14;15(22):4777. doi: 10.3390/nu15224777 (PMC10674454; doi:10.3390/nu15224777)
Supplement: Supplementary file 1 [file nutrients-15-04777-s001.zip › nutrients-2683590-supplementary.pdf]

Supplementary Materials

Supplementary S1: Search Technique

COCHRANE

ID	Search	Hits
#1	MeSH descriptor: [Crohn Disease] explode all trees	1641
#2	MeSH descriptor: [Vitamins] this term only	4153
#3	MeSH descriptor: [Micronutrients] explode all trees	5901
#4	#2 OR #3	5902
#5	#1 AND #4	28

OVID MEDLINE

Ovid MEDLINE(R) and Epub Ahead of Print, In-Process, In-Data-Review & Other Non-Indexed Citations, Daily and Versions(R) <1946 to June 14, 2021>

1 (Crohn* or (Crohn Disease or Crohn abscess or "Crohn's disease of small intestine" or Crohn Disease like Reaction or "Crohn's disease of large bowel" or Crohns disease aggravated or Crohns associated arthritis or Arthropathy in Crohn's disease or Juvenile arthritis in Crohn disease or abscess Crohn large intestine or arthritis Crohn manifestation or small intestine abscess regional enteritis or Crohn with large intestine with small intestine or abscess Crohn small intestine with large intestine)).mp. [mp=title, abstract, original title, name of substance word, subject heading word, floating sub-heading word, keyword heading word, organism supplementary concept word, protocol supplementary concept word, rare disease supplementary concept word, unique identifier, synonyms] 59406

2 limit 1 to (english language and humans) 43134

3 (inflammatory bowel disease or (Inflammatory Bowel Diseases or Inflammatory Bowel Disease Agents TC or noninfective inflammatory bowel disease)).mp. [mp=title, abstract, original title, name of substance word, subject heading word, floating sub-heading word, keyword heading word, organism supplementary concept word, protocol supplementary concept word, rare disease supplementary concept word, unique identifier, synonyms] 58001

4 limit 3 to (english language and humans) 39919

5 2 or 4 65980

6 (vitamin* or (vitamin A or Vitamins or Vitamin A EPC or Vitamin A measurement or vitamin excess vitamin A nutritional or vitamin excess vitamin K or vitamin A vitamin E or lecithin vitamin A vitamin E or vitamin A vitamin E Pill or ascorbic acid or ergocalciferol or niacinamide or tretinoin or vitamin E or retinol acetate or Administering Vitamin or D Vitamin or Retinol Activity Equivalent or folic acid or vitamin B complex or vitamin D or vitamin K or Vitamin A Deficiency or vitamin A Oral Capsule)).mp. [mp=title, abstract, original title, name of substance word, subject heading word, floating sub-heading word, keyword heading word, organism supplementary concept word, protocol supplementary concept word, rare disease supplementary concept word, unique identifier, synonyms] 374460

7 (micronutrient or (Micronutrients or "Deficiency of micronutrients")).mp. [mp=title, abstract, original title, name of substance word, subject heading word, floating sub-heading word, keyword heading word, organism supplementary concept word, protocol supplementary concept word, rare disease supplementary concept word, unique identifier, synonyms] 19265

8 6 or 7 385335

9 5 and 8 1476

OVID EMBASE

Embase <1974 to 2021 June 14>

1 (Crohn* or (Crohn Disease or Crohn abscess or "Crohn's disease of small intestine" or Crohn Disease like Reaction or "Crohn's disease of large bowel" or Crohns disease aggravated or Crohns associated arthritis or Arthropathy in Crohn's disease or Juvenile arthritis in Crohn disease or abscess Crohn large intestine or arthritis Crohn manifestation or small intestine abscess regional enteritis or Crohn with large intestine with small intestine or abscess Crohn small intestine with large intestine)).mp. [mp=title, abstract, heading word, drug trade name, original title, device manufacturer, drug manufacturer, device trade name, keyword, floating subheading word, candidate term word] 109254

2 limit 1 to (english language and humans) 88084

3 (inflammatory bowel disease or (Inflammatory Bowel Diseases or Inflammatory Bowel Disease Agents TC or noninfective inflammatory bowel disease)).mp. [mp=title, abstract, heading word, drug trade name, original title, device manufacturer, drug manufacturer, device trade name, keyword, floating subheading word, candidate term word] 98941

4 limit 3 to (english language and humans) 77831

5 2 or 4 126990

6 (vitamin* or (vitamin A or Vitamins or Vitamin A EPC or Vitamin A measurement or vitamin excess vitamin A nutritional or vitamin excess vitamin K or vitamin A vitamin E or lecithin vitamin A vitamin E or vitamin A vitamin E Pill or ascorbic acid or ergocalciferol or niacinamide or tretinoin or vitamin E or retinol acetate or Administering Vitamin or D Vitamin or Retinol Activity Equivalent or folic acid or vitamin B complex or vitamin D or vitamin K or Vitamin A Deficiency or vitamin A Oral Capsule)).mp. [mp=title, abstract, heading word, drug trade name, original title, device manufacturer, drug manufacturer, device trade name, keyword, floating subheading word, candidate term word] 466562

7 (micronutrient or (Micronutrients or "Deficiency of micronutrients")).mp. [mp=title, abstract, heading word, drug trade name, original title, device manufacturer, drug manufacturer, device trade name, keyword, floating subheading word, candidate term word] 23174

8 6 or 7 478359

9 5 and 8 4344

EBSCO CINAHL

**#5**	S1 AND S4
**#4**	S2 OR S3
**#3**	(MM "Vitamin B6 Deficiency") OR (MM "Vitamin B Deficiency+") OR "vitamin" OR (MM "Vitamin B12 Deficiency+") OR (MM "Vitamin D Deficiency+") OR (MM "Vitamin A Deficiency") OR (MM "Vitamin K Deficiency+") OR (MM "Vitamin E Deficiency") OR (MM "Riboflavin Deficiency") OR (MM "Pantothenic Acid") OR (MM "Vitamin B12 Injection (Saba CCC)") OR (MM "Thiamine Deficiency+") OR (MM "Ascorbic Acid Deficiency+") OR (MM "Vitamin K") OR (MM "Vitamin E") OR (MM "Vitamin D+")
**#2**	micronutrient*
**#1**	(MM "Crohn Disease") OR (MM "Infliximab") OR (MM "Inflammatory Bowel Diseases+") OR "crohn*" OR (MM "Ileitis+")

WEB OF SCIENCE

**#3**	#2 AND #1
**#2**	TS=(micronutrient or Micronutrients or "Deficiency of micronutrients" or vitamin or (Vitamins or vitamin A or Vitamin A EPC or vitamins deprivation or Vitamin A measurement or VITAMIN A VITAMIN D or vitamin excess vitamin A nutritional or vitamin excess vitamin K or folic acid or vitamin A vitamin E or niacinamide or lecithin vitamin A vitamin E or pantothenic acid or vitamin A vitamin E Pill or riboflavin or ascorbic acid or thiamine or ergocalciferol or Vitamin A Deficiency or vitamin K or tretinoin or vitamin E or Vitamin disease or Multivitamin preparation or D Vitamin or Avitaminosis or vitamin D or Vitamin measurement or Vitamin intake)) Indexes=SCI-EXPANDED Timespan=All years
**#1**	TI=(Crohn* or Crohn Disease or Crohn abscess or "Crohn's disease of small intestine" or Crohn Disease like Reaction or "Crohn's disease of large bowel" or Crohns disease aggravated or Cro hns associated arthritis or Arthropathy in Crohn's disease or Juvenile arthritis in Crohn disease o r abscess Crohn large intestine or arthritis Crohn manifestation or small intestine abscess region al enteritis or Crohn with large intestine with small intestine or abscess Crohn small intestine wit h large intestine) OR AB=(Crohn* or Crohn Disease or Crohn abscess or "Crohn's disease of s mall intestine" or Crohn Disease like Reaction or "Crohn's disease of large bowel" or Crohns dis ease aggravated or Crohns associated arthritis or Arthropathy in Crohn's disease or Juvenile art hritis in Crohn disease or abscess Crohn large intestine or arthritis Crohn manifestation or small intestine abscess regional enteritis or Crohn with large intestine with small intestine or abscess Crohn small intestine with large intestine) Indexes=SCI-EXPANDED Timespan=All years

Supplementary S2: Study quality checklist

1) Was micronutrient insufficiency or status a primary stated objective of the study?
2) Was there exclusion of or adequate reporting of confounding factors such as:
   a. Serum inflammatory markers?
   b. Previous bowel resections?
   c. Supplement use?
3) If not an exclusion or separate analysis, were the following characteristics of the cohort representative of a current outpatient CD population? (in a country with access to biological medications)
   a. Inflammatory state

   b. Previous bowel resections
   c. Supplement use

4) Were subjects recruited in a clearly defined, acceptable way to minimise bias? (Classed Yes / Unclear / No)
5) Was the micronutrient status recorded prospectively?
6) Were micronutrient data reported in a clear way (i.e., not on graphs alone, with defined cut off for deficiency prevalence studies, and with appropriate statistical tests for HC comparison studies)?
7) If CD subjects in clinical remission were reported separately or this was an inclusion criterion, was remission clearly defined?
8) If those in remission were not reported separately, was the percentage of subjects in clinical remission clearly defined?
9) Overall quality? (Low / Medium / High)

Supplementary S3: Data extracted from eligible studies

Vitamin A (Retinol)

**Vitamin A** Author [main text ref]	Year	N CD (HC)	P/R	Data on % Deficiency in CD	CD vs HC	Separate reporting of remission? Definition of remission or Cohort characteristics (CCh)	Prevalence of Deficiency In CD remission group or if not separately reported in CD group<br><br>Difference vs HC	Supports Vit A deficiency being present in CD remission	Supports Vit A status being altered in Cd remission in comparison with healthy controls (HC)	Exploration of cause Subgroup analysis or Special tests	Overall Quality (see table 2)
Schoelmerich [21]	1985	54 (19)	P		X	Yes, reports “mildly active” CDAI <150, mild laboratory changes no active symptoms and “inactive” with CDAI<150, no laboratory changes or active symptoms	Vit A lower in mildly active CD group vs HC 45.5+/-16.5 µg/dl vs 67.3+/-9.1 (p<0.01)<br><br>Vit A among “inactive” CD, vs HC 67.3+/-9.1 vs 69.9+/-16 (NS)	Not reported	Yes, but in the group with CDAI<150 and mild symptoms only	Strong negative correlation with CDAI *R* -0.6196 *p* <0.001	Medium
Geerling [16]	1998	32 (32)	P	X	X	No separate remission group<br><br>(CCh): Median CDAI 139, 53% remission (CDAI <150)	28% (0.9µmol/L)<br><br>No CD diff v HC 2.6 +/-1.3 µmol/L vs 2.5 +/- 0.06 µmol/L *NS*	Yes	No	Intake of Vitamin A assessed with FFQ: Less than RDA in 69% CD and 91% HC. Intake not different<br><br>No reported intake vs serum correlation analyses. Nil analysis Vit A vs disease activity	Medium

**Vitamin A** Author [main text ref]	Year	N CD (HC)	P/R	Data on % Deficiency in CD	CD vs HC	Separate reporting of remission? Definition of remission or Cohort characteristics (CCh)	Prevalence of Deficiency In CD remission group or if not separately reported in CD group<br><br>Difference vs HC	Supports Vit A deficiency being present in CD remission	Supports Vit A status being altered in Cd remission in comparison with healthy controls (HC)	Exploration of cause Subgroup analysis or Special tests	Overall Quality (see table 2)
Geerling [17]	1999	62 (70)	P		X	Yes<br><br>(defined by Van Hees <150)	No prevalence<br><br>Comparison: Vitamin A in CD remission vs HC not different.<br><br>Vit A lower in active vs remission CD	Not reported	No	No assessment of intake<br>Median serum Vit A in 12 with active CD (CDAI >150) compared with 50 CD remission- *NS*<br><br>Vitamin A in the same individuals who were in remission and relapsed was significantly lower in relapse	Medium
Genser [24]	1999	24 (33)	P		X	Remission not reported separately<br>CCh:<br>87% in remission (CDAI <150)	Retinol in CD 44.69+/-9.18 µg/dl Vs 51.44+/-12.71 in HC *NS*	Not clear / reported	No	The total radical-trapping antioxidant potential (TRAP) lower in CD vs HC	Medium

**Vitamin A** Author [main text ref]	Year	N CD (HC)	P/ R	Data on % Deficiency in CD	CD vs HC	Separate reporting of remission? Definition of remission or Cohort characteristics (CCh)	Prevalence of Deficiency In CD remission group or if not separately reported in CD group<br><br>Difference vs HC	Supports Vit A deficienc y being present in CD remissio n	Supports Vit A status being altered in Cd remission in comparison with healthy controls (HC)	Exploration of cause Subgroup analysis or Special tests	Overall Quality (see table 2)
Geerling [18]	2000	23 (23)	P		X	Remission not reported separately<br>CCh:<br>87% in remission (CDAI <150)<br>Mean (+/-SD) CDAI 96.9+/-66.5	Mean +/-SD in CD: 2.2+/-0.8<br>Vs<br>HC: 2.4+/-0.6<br>*NS*	Not clear / reported	No	Intake of Vitamin A assessed with FFQ and diary<br>CD vs HC *NS*<br>No reported intake vs serum correlation analyses.	High
Wendland [28]	2000	34 (37)	P		X	Not for comparison of Vit A with HC<br><br>CCh:<br>70% in remission (CDAI <150)<br>CDAI Mean +/- SEM<br>141+/-18.6	Retinol (mean +/- SEM) CD:<br>1.84+/-0.11<br>HC:1.96+/-0.07 *NS*<br><br>**mean +/- SEM in CDr (CDAI<150)<br>1.92+/-0.11. n HC comparison	Not reported	No	Intake not reduced vs HC n 7d food diary.<br>No correlation analyses of intake vs plasma.<br>Vit A correlated to serum orosomucoid (-), but not to CDAI or lipid peroxidation (as measured by breath pentanes, which was lower in CD vs HC)	Medium

**Vitamin A** Author [main text ref]	Year	N CD (HC)	P/R	Data on % Deficiency in CD	CD vs HC	Separate reporting of remission? Definition of remission or Cohort characteristics (CCh)	Prevalence of Deficiency In CD remission group or if not separately reported in CD group<br><br>Difference vs HC	Supports Vit A deficiency being present in CD remission	Supports Vit A status being altered in Cd remission in comparison with healthy controls (HC)	Exploration of cause Subgroup analysis or Special tests	Overall Quality (see table 2)
D'Odorico [26]	2001	33 (386)	?		X	Not for comparison of Vit A with HC<br><br>CCh:<br>70% in remission (CDAI <150)	Retinol lower in CD than HC 1.67+/-0.1µmol/L Vs 2.7+/0.07µmol/L *P*<0.0001<br><br>Vit A- active vs remission NS diff 1.55+/-0.5 vs 1.69+/-0.1	Not clear / reported	Yes	Intake compared between CD and HC by FFQ estimated macronutrient, fruit and veg. CD intake of energy, protein and Veg lower than HC<br><br>Vit A in remission (n=23) vs active (n=10) *NS*<br><br>BMI <20; Vit A 1.3+/-0.13 vs >20 1.8+/-0.07 *P* 0.017	Low

**Vitamin A** Author [main text ref]	Year	N CD (HC)	P/ R	Data on % Deficiency in CD	CD vs HC	Separate reporting of remission? Definition of remission or Cohort characteristics (CCh)	Prevalence of Deficiency In CD remission group or if not separately reported in CD group<br><br>Difference vs HC	Supports Vit A deficienc y being present in CD remissio n	Supports Vit A status being altered in Cd remission in comparison with healthy controls (HC)	Exploration of cause Subgroup analysis or Special tests	Overall Quality (see table 2)
Vagianos [34]	2007	84	P	X	No	No separate remission CD group<br><br>CCh:<br>61% in remission (HBI<5)	1.23% (<360μg/L)	Yes	Not reported	Intake assessed with FFQ then 4-day food diary then compared vs adequacy (defined as <66% dietary reference value (DRV))<br>Vit A intake inadequate in 30% CD Spearman Correlation of Intake / 100kcal vs serum Vit A *NS*	Medium

**Vitamin A** Author [main text ref]	Year	N CD (HC)	P/ R	Data on % Deficiency in CD	CD vs HC	Separate reporting of remission? Definition of remission or Cohort characteristics (CCh)	Prevalence of Deficiency In CD remission group or if not separately reported in CD group<br><br>Difference vs HC	Supports Vit A deficiency being present in CD remission	Supports Vit A status being altered in Cd remission in comparison with healthy controls (HC)	Exploration of cause Subgroup analysis or Special tests	Overall Quality (see table 2)
Soares-Mota [46]	2015	38 (33)		X	X	Remission not reported separately<br><br>CCh:<br>89%34/38 in remission (HBI<5)	CD: 29% deficient (<1.05µmol/L)<br>Vs<br>HC: 12% p<0.005<br><br>Retinol dose response test (increase >20%) 37% CD +ve vs 12% HC (reflects liver store)	Yes	Yes	Retinol dose response (RDR) was performed to check adequacy of hepatic stores<br>Positive (increase of 20% 5 hours after 2500iU PO retinol palmitate) in 37% of CD vs 12% HC<br><br>Comparison of characteristics of those with and without low serum Vit A found lower BMI and Fat Mass (DEXA) in those with low Vit A	Medium

**Vitamin A** Author [main text ref]	Year	N CD (HC)	P/ R	Data on % Deficiency in CD	CD vs HC	Separate reporting of remission? Definition of remission or Cohort characteristics (CCh)	Prevalence of Deficiency In CD remission group or if not separately reported in CD group<br><br>Difference vs HC	Supports Vit A deficiency being present in CD remission	Supports Vit A status being altered in Cd remission in comparison with healthy controls (HC)	Exploration of cause Subgroup analysis or Special tests	Overall Quality (see table 2)
MacMaster [20]	2021	59		X		All in clinical remission (HBI) No separate CD<br><br>53% in biochem remission (BCR) (FCP < 250, CRP <10 albumin >35) (not report separately)	2% deficiency<br><br>1.0μmol/ml	Yes	Not reported	Relationship between albumin (ALB), faecal calprotectin (FCP) and c-reactive protein (CRP) explored with Spearman's RHO. Only ALB correlated (rho=0.20 p =0.061),<br><br>No exploration of phenotypic predictors of deficiency done.<br><br>Survival analysis of flare in subsequent 12 months found no relationship	Medium

Beta Carotene

ß-**Carotene** Author [main text ref number]	Year	N CD HC	P/R	Data on % Deficiency in CD	CD vs HC	Separate reporting of remission? Definition of remission or Cohort characteristics (CCh)	Prevalence of Deficiency In CD remission group or if not separately reported in CD group Difference vs HC	Supports ß-Carotene deficiency being present in CD remission	Supports ß-Carotene status being altered in Cd remission in comparison with healthy controls (HC)	Subgroup analysis or exploration of cause or Special tests	Overall Quality (see table 2)
Geerling [16]	1998	32 (32)	P	X	X	No separate remission group<br><br>(CCh):<br>Median CDAI 139, 53% remission (CDAI <150)	Prevalence of deficiency (<0.4µmol/L): 100%<br>ß-carotene lower CD: mean(+/-SD) 0.98+/-0.91 µmol/l Vs 2.33+/-0.70 in HC (P <0.001)	Yes	Yes	Intake of ß-Carotene assessed with FFQ: Intake not different CD vs HC. No RDA for ß-Carotene.<br>Ratio of ß-Carotene to lipids lower in CD<br>Nil analysis ß-Carotene vs disease activity	Mediu m
Geerling [18]	1999	62 (70)	P		X	Yes, inclusion criteria for study<br><br>(defined by Van Hees <150)	No prevalence<br><br>ß-Carotene lower in CD inactive: Median (IQR)<br>1.74 (1.28-2.45)<br>vs 2.24(1.80-2.8)<br>(P 0.003)	Not reported	Yes	Intake of ß-Carotene assessed with FFQ but only comparison between CD active and CD remission NS<br><br>Serum ß-Carotene in CDa lower than CDr 1.04 (0.71-1.54) vs 1.74(1.28-2.83)	Mediu m
Genser [24]	1999	24 (33)	P		X	Remission not reported separately (CCh):<br>87% in remission (CDAI <150)	No prevalence data<br>ß-carotene lower in CD:<br>16.20+/-12.42 µg/dl Vs 34.74+/-18.84 in HC (P <0.0001)	Not reported	Yes	The total radical-trapping antioxidant potential (TRAP) lower in CD vs HC	Mediu m

**ß-Carotene** Author [main text ref number]	Year	N CD HC	P/R	Data on % Deficiency in CD	CD vs HC	Separate reporting of remission? Definition of remission or Cohort characteristics (CCh)	Prevalence of Deficiency In CD remission group or if not separately reported in CD group Difference vs HC	Supports ß-Carotene deficiency being present in CD remission	Supports ß-Carotene status being altered in Cd remission in comparison with healthy controls (HC)	Subgroup analysis or exploration of cause or Special tests	Overall Quality (see table 2)
Geerling [18]	2000	23 (69)	P		X	Remission not reported separately (CCh):<br>87% in remission (CDAI <150)<br>Mean (+/-SD) CDAI 96.9+/-66.5	Prevalence not reported<br>Mean +/-SD in CD: 2.2+/-1.3<br>Vs<br>HC: 1.8+/-0.7<br>*NS*	Not reported	No	Intake of ß-Carotene assessed with FFQ and diary<br>CD vs HC *NS*<br>No reported intake vs serum correlation analyses.	Medium
Wendland [28]	2001	34 (37)	P		X	Not for comparison of ß-Carotene with HC<br>CCh:<br>70% in remission (CDAI <150)<br>CDAI Mean +/- SEM 141+/-18.6	ß-Carotene (mean +/-SEM) lower in CD: 0.36+/-0.05 than in HC:0.52+/-0.05 *P*<0.05<br><br>**mean +/- SEM in CDr (CDAI<150) 0.41+/-0.07	Not reported	Yes for all CD vs HC, but CDr vs HC not reported	Intake not reduced vs HC on 7d food diary.<br>No correlation analyses of intake vs plasma.<br>Serum concentration correlated to CDAI (-), but not to serum orosomucoid or lipid peroxidation (as measured by breath pentanes, which was lower in CD vs HC)	Medium

**ß-Carotene** Author [main text ref number]	Year	N CD HC	P/R	Data on % Deficiency in CD	CD vs HC	Separate reporting of remission? Definition of remission or Cohort characteristics (CCh)	Prevalence of Deficiency In CD remission group or if not separately reported in CD group Difference vs HC	Supports ß-Carotene deficiency being present in CD remission	Supports ß-Carotene status being altered in Cd remission in comparison with healthy controls (HC)	Subgroup analysis or exploration of cause or Special tests	Overall Quality (see table 2)
D'Odorico [26]	2001	23	P		X	Not for comparison of ß-carotene with HC<br><br>CCh:<br>70% in remission (CDAI <150)	Prevalence not reported<br><br>ß-carotene (Mean +/-SD) lower in CD: 0.41+/-0.08µmol/L Vs HC 0.83+/-0.03<br>P <0.0001	Not clear / reported	Yes	Intake compared between CD and HC by FFQ estimated macronutrient, fruit and veg. CD intake of energy, protein and Veg lower than HC<br>ß-Carotene lower in active (n=10) 0.46+/-0.09 vs remission (n=23) 0.11+/-0.04 *P*<0.05	Low

**ß-Carotene** Author [main text ref number]	Year	N CD HC	P/R	Data on % Deficiency in CD	CD vs HC	Separate reporting of remission? Definition of remission or Cohort characteristics (CCh)	Prevalence of Deficiency In CD remission group or if not separately reported in CD group Difference vs HC	Supports ß-Carotene deficiency being present in CD remission	Supports ß-Carotene status being altered in Cd remission in comparison with healthy controls (HC)	Subgroup analysis or exploration of cause or Special tests	Overall Quality (see table 2)
Filippi [19]	2006	54 (25)	P	X		All in remission<br><br>CDAI<br>All subjects needed 3/12 (CDAI <150)<br><br>Mean CRP 6.6-/-0.8mg/dl	Prevalence (no definition provided) 20% (estimated from graph)<br><br>Biochemical analysis of HC not reported (whether as prevalence of deficiency or results)<br><br>CD Subjects' mean or median ß-carotene Not reported	Yes	Not clear / reported	Mean Intake of ß-carotene assessed with 3-day food diary in CD and compared vs HC:<br>CD lower than HC:<br>Females:<br>1461.76+/-339.98 vs 3254.36+/-569.76<br>$P<0.005$<br>Males:<br>1720.84+/-314.74 vs 2933.00+/-494.76<br>$P<0.05$ men<br>Both M and F CD intake <French RDA (2100)	Low

**ß-Carotene** Author [main text ref number]	Year	N CD HC	P/R	Data on % Deficiency in CD	CD vs HC	Separate reporting of remission? Definition of remission or Cohort characteristics (CCh)	Prevalence of Deficiency In CD remission group or if not separately reported in CD group Difference vs HC	Supports ß-Carotene deficiency being present in CD remission	Supports ß-Carotene status being altered in Cd remission in comparison with healthy controls (HC)	Subgroup analysis or exploration of cause or Special tests	Overall Quality (see table 2)
Vagianos [34]	2007	84	P	X		No separate remission CD group<br><br>CCh:<br>61% in remission (HBI<5)	29.3% (<1.1µmol/L)	Yes	Not clear / reported	Intake assessed with FFQ then 4-day food diary then compared vs adequacy (defined as <66% dietary reference value (DRV))<br>Vit A intake inadequate in 30% CD<br><br>Spearman Correlation of Vit A Intake / 100kcal vs serum ß-carotene weakly correlated *r* 0.27 P0.02	Mediu m

Vitamin D (25-OH D3)

**Vitamin D (25 OH D)** Author [main text ref number]	Year	N CD (HC)	P/ R	Data on % Deficiency in CD	CD vs HC	Separate reporting of remission? Definition of remission or Cohort characteristics (CCh)	Prevalence of Deficiency In CD remission (CDr) group or if not separately reported in CD group Difference vs HC	Supports Vit D deficiency being present in CD remission	Supports Vitamin D status being altered in comparison with healthy controls (HC)	Subgroup analysis or exploration of cause Exploration of consequences Special tests	Overall Quality (see table 2)
Duggan [29]	2004	44 (44)	P		X	All in remission- defined as the Absence of GI symptoms	Prevalence not reported<br><br>Mean (SD) 25-OHD mmol/L CD: 75.0 (28.7) vs HC: 105.3 (55.5) *P* < 0.01	Not reported	Yes	Mean intake of Vitamin D in CD and HC not different	Medium
Tajika [30]	2004	33 (15)	?	X	X	No separate reporting of remission. CCh: Mean CDAI 84.1 44.2 (range, 12.6–182.7)	27.3% of CD deficient (<10ng/ml) Mean (SD) 25-OHD CD: 15.2+/-6.5 vs HC 16.9 +/-5.2 *NS*	Yes	No	Strong relationship between 25OH-D level and both disease duration (r 0.46, *P* 0.00)3 and CDAI (*r* 0.44 *P* 0.005)	High
McCarthy [31]	2005	44 (66)	P	X	X	All 44 in remission at baseline (defined by both the absence of gastrointestinal symptoms and the requirement for corticosteroids)	18.2% of CD deficient (<50nmol/L) Vs 4.5% of HC Mean (SD) 25-OHD CD: 75.0 (28.7) vs HC 105.3 (55.5) *P<0.0001*	Yes	Yes	Vit D intake assessed by FFQ. No difference CD vs HC- no correlation analysis of intake vs serum Urinary terminal n-peptides elevated in CD vs HC but exploration vs Vit D	High

**Vitamin D (25 OH D)** Author [main text ref number]	Year	N CD (HC)	P/ R	Data on % Deficiency in CD	CD vs HC	Separate reporting of remission? Definition of remission or Cohort characteristics (CCh)	Prevalence of Deficiency In CD remission (CDr) group or if not separately reported in CD group Difference vs HC	Supports Vit D deficiency being present in CD remission	Supports Vitamin D status being altered in comparison with healthy controls (HC)	Subgroup analysis or exploration of cause Exploration of consequences Special tests	Overall Quality (see table 2)
Filippi [19]	2006	54 (25)	P	X		All in remission<br><br>CDAI<br>All subjects needed 3/12 (CDAI <150)<br><br>Mean CRP 6.6-/-0.8mg/dl	Prevalence (no definition provided) 10% of CDr deficient (est. from graph)<br>Biochemical analysis of HC not reported (whether as prevalence of deficiency or results)<br>CDr Subjects' mean or median 25-OHD Not reported	Yes	Not clear / reported	Mean Intake of 25-OH D assessed with 3-day food diary in CD and compared vs HC in male and female separately Intake in CD f (n=28) vs HC f (n=16) and CD m (n=26) vs HC m (n=9) *NS*<br>Mean intake in CD and HC m and f <French RDA (5µg/day)	Low
Gilman [32]	2006	58	P	X		Remission not reported separately<br><br>48/58 (82.8%) in remission (No GI symptoms)	19% Deficient (<50nmol/L) at baseline (late Summer)	Yes	Not clear / reported	Multiple regression found 25 OHD + assoc. with supplements, - associated with smoking. Study had winter check showing 45% deficient, with SB involvement also predictive	High

**Vitamin D (25 OH D)** Author [main text ref number]	Year	N CD (HC)	P/R	Data on % Deficiency in CD	CD vs HC	Separate reporting of remission? Definition of remission or Cohort characteristics (CCh)	Prevalence of Deficiency In CD remission (CDr) group or if not separately reported in CD group Difference vs HC	Supports Vit D deficiency being present in CD remission	Supports Vitamin D status being altered in comparison with healthy controls (HC)	Subgroup analysis or exploration of cause Exploration of consequences Special tests	Overall Quality (see table 2)
Vagianos [34]	2007	84	P	X		No separate remission CD group<br><br>CCh:<br>61% in remission (HBI<5)	45.8% (<50nmol/L) at inter time-point	Yes	Not clear / reported	Intake assessed with FFQ then 4-day food diary inc supplements then compared vs adequacy (defined as <66% dietary reference value (DRV)) Intake inadequate in 38% CD<br>Spearman Correlation of Vit D Intake / 100kcal vs serum 25OH D strongly correlated *r* 0.41 *P*<0.001	Medium
Nakajima [37]	2011	47 (41)	P		X	No separate remission group<br><br>Cohort characteristics Mean (+/-SD) CDAI: 107.6+/-88.0 50%	Prevalence not reported<br><br>25 OH-D reported as significantly higher in CD than HC (P<0.05) but values only displayed on graph	Not reported	Yes	25-OH Not correlated with to CDAI.	Low

**Vitamin D (25 OH D)** Author [main text ref number]	Year	N CD (HC)	P/R	Data on % Deficiency in CD	CD vs HC	Separate reporting of remission? Definition of remission or Cohort characteristics (CCh)	Prevalence of Deficiency In CD remission (CDr) group or if not separately reported in CD group Difference vs HC	Supports Vit D deficiency being present in CD remission	Supports Vitamin D status being altered in comparison with healthy controls (HC)	Subgroup analysis or exploration of cause Exploration of consequences Special tests	Overall Quality (see table 2)
Nic Suibhne [38]	2012	81 (70)	P	X	X	Remission not reported separately No separate remission CD group<br><br>CCh:<br>(median CDAI 92.09 (IQR 39-164)<br>CRP 3.85 (2.9-9.6))	63% in CD (<50nmnol/L)<br>Vs 55% HC (NS)<br><br>Mean (+/-SD) level CD 47.76+/-27.27 vs HC 51.86+/-24.53 (NS)	Yes	No	Daily Intake from diet alone assessed FFQ. Lower in CD than HC. 1.0µg/day vs 1.6µg/day *P*<0.001<br>43% Cd took supplement vs 16% HC<br>Regression analysis identified winter season, smoking and longer disease duration as associations. BMI, CRP, disease location age and gender surgery and steroid use did not contribute	High

**Vitamin D (25 OH D)** Author [main text ref number]	Year	N CD (HC)	P/ R	Data on % Deficiency in CD	CD vs HC	Separate reporting of remission? Definition of remission or Cohort characteristics (CCh)	Prevalence of Deficiency In CD remission (CDr) group or if not separately reported in CD group Difference vs HC	Supports Vit D deficiency being present in CD remission	Supports Vitamin D status being altered in comparison with healthy controls (HC)	Subgroup analysis or exploration of cause Exploration of consequences Special tests	Overall Quality (see table 2)
Garg [41]	2013	40 (23)	P	X	X	Remission not reported separately No separate remission CD group<br><br>CCh:<br>Median (range) HBI 3 (0-16)<br>Remission defined by CRP <5 and FCP<150 in 55%	23% in CD (<50nmol/L)<br>Vs<br>26% in HC<br>*NS*<br><br>Mean (CI) 25OHD In CD: 70 (61-78)<br>Vs.<br>HC: 66 (56-76)<br>*NS*	Yes	No	25 OHD inversely moderately correlated to faecal calprotectin (but not CRP)<br>Pearson's r =-0.35, P 0.040),	Medium
Grunbaum [42]	2013	34	P	X	X	Remission not reported separately<br><br>CCh:<br>79.% Remission (HBI<5)<br>20.6% "mildly active" (HBI>5)	29.4% in CD (<50nmol/L)<br>12.7% in Healthy family, 22.9% in HC<br><br>Mean (+/-SD) 25OHD in CD 71.1+/-31.1 vs family 32.2+/-34.2 and HC 68.3+/-27.6<br><br>p value for CD comparison vs family and HC not reported	Yes	Not clear / reported	Nil separate analysis of 25 OH D correlates in IBD cases.	Low

**Vitamin D (25 OH D)**<br>Author [main text ref number]	Year	N CD (HC)	P/ R	Data on % Deficiency in CD	CD vs HC	Separate reporting of remission?<br>Definition of remission<br>or Cohort characteristics (CCh)	Prevalence of Deficiency<br>In CD remission (CDr) group or if not separately reported in CD group<br>Difference vs HC	Supports Vit D deficiency being present in CD remission	Supports Vitamin D status being altered in comparison with healthy controls (HC)	Subgroup analysis or exploration of cause<br>Exploration of consequences<br>Special tests	Overall Quality (see table 2)
Jorgensen [43]	2013	182	P		X	Comparative statistics with HC only performed with whole CD cohort<br><br>CCh: 102/182 in remission (CDAI <150)<br><br>mean 25 OHD of CDAI<150 reported separately	Prevalence not reported<br><br>Median 25OHD in CD:<br>69nmol/L<br>Vs<br>HC: 65nmol/L<br>*NS*<br><br>Median Vit D of 103 in remission 64nmol/L	Not clear / reported	No	25 OH D compared between CDAI defined remission, mild moderate and severe.<br>Inversely related: remission 64, mild: 49, moderately active 21<br>(Kruskal's and spearman's rho <0.01). similar, less pronounced trend with CRP. Higher supplement use noted in CD (44%) than HC (10%)	Low
Kini [44]	2014	32	P	X		Remission not reported separately<br><br>CCh:<br>Mean CDAI Winter (only data extracted) 103.9 (Range 10-279, SD 76.9)	76% deficient in Winter (<50nmol/L)	Yes	Not reported	No correlation was found between seasonal serum Vitamin D levels and CDAI	Medium

**Vitamin D (25 OH D)** Author [main text ref number]	Year	N CD (HC)	P/R	Data on % Deficiency in CD	CD vs HC	Separate reporting of remission? Definition of remission or Cohort characteristics (CCh)	Prevalence of Deficiency In CD remission (CDr) group or if not separately reported in CD group Difference vs HC	Supports Vit D deficiency being present in CD remission	Supports Vitamin D status being altered in comparison with healthy controls (HC)	Subgroup analysis or exploration of cause Exploration of consequences Special tests	Overall Quality (see table 2)
Raffner Basson [48]	2016	44	P	X		Yes, Remission (HBI<5) reported separately	14% of CDr deficient (≤20 ng/mL)<br>30% of CDr insufficient (≤30 ng/mL)<br>Vs.<br>53% of HC deficient<br>And<br>89% of HC insufficient	Yes	No, prevalence of deficiency higher in HC than CDr (no direct comparison of 25OHD levels)	Primary aim of study was to compare the prevalence of deficiency as grouped by HBI. This demonstrated progressive increase in deficiency at HBI 5-7 (28%) and ≥8 (45%), respectively.	Medium
Frigstad [50]	2017	230	P	X		Remission not reported separately<br><br>CCh:<br>53% in remission (HBI<5)<br>Median HBI 4	53% insufficient (<50nmol/L)<br>8% severely deficient (<25nmol/L)	Yes		Risk Factors for low 25OHD in multivariate analysis:<br>HBI>8:<br>OR 3.06 (95% CI 1.27-7.35)<br>BMI >25<br>OR 1.95 (95% CI 1.03-3.70) (smoking, age gender, resections, disease duration pr behaviour, medications not associated)	High

**Vitamin D (25 OH D)** Author [main text ref number]	Year	N CD (HC)	P/ R	Data on % Deficiency in CD	CD vs HC	Separate reporting of remission? Definition of remission or Cohort characteristics (CCh)	Prevalence of Deficiency In CD remission (CDr) group or if not separately reported in CD group Difference vs HC	Supports Vit D deficiency being present in CD remission	Supports Vitamin D status being altered in comparison with healthy controls (HC)	Subgroup analysis or exploration of cause Exploration of consequences Special tests	Overall Quality (see table 2)
Cavaziel [51]	2018	99	P	X	No	Remission not reported separately<br><br>CCh:<br>70% in clinical remission (HBI<5)	58% (<50nmol/L)	Yes	Not reported	In a linear regression model adjusted for age, gender, and BMI, a significant inverse association of C-reactive protein (CRP) ($p = 0.031$) and faecal calprotectin (FC) ($p = 0.025$) with 25-OH-D3 levels	Medium
Branco [52]	2019	106	P	X	No	Remission not reported separately<br>CCh:<br>71.9% in remission (HBI<5)<br>Mean(+/-SD) HBI: 2.7+/-3	90.8% Insufficient (21-29ng/ml)<br>75% Deficient (<20ng/ml)<br>24% Severe deficiency (<10ng/ml)	Yes	Not reported	25-OH D negatively correlated with CRP HBI and age	High

**Vitamin D (25 OH D)** Author [main text ref number]	Year	N CD (HC)	P/R	Data on % Deficiency in CD	CD vs HC	Separate reporting of remission? Definition of remission or Cohort characteristics (CCh)	Prevalence of Deficiency In CD remission (CDr) group or if not separately reported in CD group Difference vs HC	Supports Vit D deficiency being present in CD remission	Supports Vitamin D status being altered in comparison with healthy controls (HC)	Subgroup analysis or exploration of cause Exploration of consequences Special tests	Overall Quality (see table 2)
Olmedo-Martín [55]	2019	150 (104 remission)	P	X		Yes HBI <5	23.1% of 104 CDr deficient (<20ng/ml)	Yes	Not reported	Proportion of CD subjects with deficiency higher in Winter than summer: 48.5% vs 21.4%; *P*<0.001<br><br>When grouped by 25 OHD deficient / insufficient / adequate statistically significant difference FCP between groups (104.8 vs 84 vs 47.2) ANOVA *P* 0.002 and higher % with HBI active disease in deficiency vs inadequate vs adequate (34%, 24% and 7%) P <0.001	High

**Vitamin D (25 OH D)** Author [main text ref number]	Year	N CD (HC)	P/ R	Data on % Deficiency in CD	CD vs HC	Separate reporting of remission? Definition of remission or Cohort characteristics (CCh)	Prevalence of Deficiency In CD remission (CDr) group or if not separately reported in CD group Difference vs HC	Supports Vit D deficiency being present in CD remission	Supports Vitamin D status being altered in comparison with healthy controls (HC)	Subgroup analysis or exploration of cause Exploration of consequences Special tests	Overall Quality (see table 2)
Zhao [56]	2019	21 (120)	P		X	Yes HBI <5	CDr: 25 OHD mean+/-SD 13.97+/-5.61 µmol/l<br><br>Vs 12.96+/-5.18 in HC *NS*	Not reported	No	Mean 25 OHD of CDr, compared with mild moderate and severe (by HBI) 25-OHD in CDr lower than moderate or severe CD (12.18+/-3.69 vs 9.21+/-3.26 vs 7.58+/-3.81, respectively) *P* <0.05	Low
Domislovic [57]	2020	123		X		Remission not reported separately<br><br>CCh: 100/123 (81.3%) in remission (HBI<5)	61.9% (<50nmol/L)	Yes	Not clear / reported	25 OH-D lower if ileal involvement Higher in those with ileal or ileocolonic resections, on anti TNF or on a supplement. Mean 25 OH D not different in active vs inactive (HBI), if on corticosteroids, on immunomodulator or colonic resections	Low

**Vitamin D (25 OH D)** Author [main text ref number]	Year	N CD (HC)	P/ R	Data on % Deficiency in CD	CD vs HC	Separate reporting of remission? Definition of remission or Cohort characteristics (CCh)	Prevalence of Deficiency In CD remission (CDr) group or if not separately reported in CD group Difference vs HC	Supports Vit D deficiency being present in CD remission	Supports Vitamin D status being altered in comparison with healthy controls (HC)	Subgroup analysis or exploration of cause Exploration of consequences Special tests	Overall Quality (see table 2)
MacMaster [20]	2021	59	P	X		All in clinical remission (HBI)<br>No separate CD<br><br>53% in biochem remission (BCR) (FCP < 250, CRP <10 albumin >35) (not report separately)	34%<br><br>25nmol/L	Yes	Not clear / reported	Relationship between albumin, FCP and crp explored with Spearman's RHO. No correlation.<br><br>Exploration of phenotypic predictors of deficiency did not report any predictors<br><br>Survival analysis of flare in subsequent 12 months found no relationship	Medium

Vitamin E

**Vitamin E** Author [main text ref number]	Year	N CD (HC)	P/R	Data on % Deficiency in CD	CD vs HC	Separate reporting of remission? Definition of remission or Cohort characteristics (CCh)	Prevalence of Deficiency In CD remission group or if not separately reported in CD group Difference vs HC	Supports Vit E deficiency being present in CD remission	Supports Vit E status being altered in Cd remission in comparison with healthy controls (HC)	Exploration of cause Subgroup analysis or Special tests	Overall Quality (see table 2)
Geerling [16]	1998	32 (32)	P	X	X	No separate remission group<br><br>(CCh):<br>Median CDAI 139, 53% remission (CDAI <150)	45% (from graph) (<14µmol/L):<br><br>Vit E lower CD: mean(+/-SD) 29.2+/-10.7 µmol/l<br>Vs<br>34.8+/-8.6 in HC<br>(*P* <0.05)	Yes	Yes	Intake of Vit E not among micronutrients assessed<br>Ratio of Vit E to serum lipids and cholesterol same in CD to HC suggesting lower cholesterol may contribute to lower Vit E in CD	Medium
Geerling [17]	1999	62 (70)	P		X	Yes, mean of 50 Cd remission reported<br><br>(defined by Van Hees <150)	Prevalence of deficiency not reported<br><br>Median (IQR) in CD remission: 29.0 (23.8-34.0) µmol/l<br>Vs 31.0 (27.0-38.3) µmol/l in HC<br>P 0.05	Not clear / reported	Yes	Intake of assessed with FFQ but only comparison between CD active and CD remission *NS*<br><br>Serum Vit E in CDa vs CDr *NS*<br><br>Exploratory correlation revealed Vit E correlated with PUFA	Medium

**Vitamin E** Author [main text ref number]	Year	N CD (HC)	P/R	Data on % Deficiency in CD	CD vs HC	Separate reporting of remission? Definition of remission or Cohort characteristics (CCh)	Prevalence of Deficiency In CD remission group or if not separately reported in CD group Difference vs HC	Supports Vit E deficiency being present in CD remission	Supports Vit E status being altered in Cd remission in comparison with healthy controls (HC)	Exploration of cause Subgroup analysis or Special tests	Overall Quality (see table 2)
Genser [24]	1999	24 (33)	P		X	Remission not reported separately (CCh): 87% in remission (CDAI <150)	Vit E in CD: 1.07 mg/dl +/- 0.23 Vs HC: 1.07+/-0.28 *NS*	Not reported	No	The total radical-trapping antioxidant potential (TRAP) lower in CD vs HC	Medium
Geerling [18]	2000	23	P		X	Remission not reported separately (CCh): 87% in remission (CDAI <150) Mean (+/-SD) CDAI 96.9+/-66.5	Prevalence of deficiency not reported<br><br>Mean +/- SD in CD: 30.4+/-8.7µmol/L Vs HC: 28.8+/5.8µmol/L NS	Not clear / reported	No	Nil assessment of intake<br><br>Vit E correlated to serum cholesterol	High

**Vitamin E** Author [main text ref number]	Year	N CD (HC)	P/R	Data on % Deficiency in CD	CD vs HC	Separate reporting of remission? Definition of remission or Cohort characteristics (CCh)	Prevalence of Deficiency In CD remission group or if not separately reported in CD group Difference vs HC	Supports Vit E deficiency being present in CD remission	Supports Vit E status being altered in Cd remission in comparison with healthy controls (HC)	Exploration of cause Subgroup analysis or Special tests	Overall Quality (see table 2)
Wendland [28]	2001	34	P		X	Not for comparison of Vit A with HC CCh: 70% in remission (CDAI <150) CDAI Mean +/- SEM 141+/-18.6	Vit E (mean +/- SEM) CD: 24.1+/-1.1 HC:22.7+/-0.62 *NS* **mean +/- SEM in CDr (CDAI<150) 23.3+/-1.23	Not reported	No	Intake not reduced vs HC on 7-day food diary. No correlation analyses of intake vs plasma. Serum concentration not correlated to CDAI, serum orosomucoid or lipid peroxidation (as measured by breath pentanes, which was lower in CD vs HC)	Medium

**Vitamin E** Author [main text ref number]	Year	N CD (HC)	P/R	Data on % Deficiency in CD	CD vs HC	Separate reporting of remission? Definition of remission or Cohort characteristics (CCh)	Prevalence of Deficiency In CD remission group or if not separately reported in CD group Difference vs HC	Supports Vit E deficiency being present in CD remission	Supports Vit E status being altered in Cd remission in comparison with healthy controls (HC)	Exploration of cause Subgroup analysis or Special tests	Overall Quality (see table 2)
D'Odorico [26]	2001	33	P		X	Not for comparison of Vit A with HC<br><br>CCh:<br>70% in remission (CDAI <150)	Prevalence of deficiency not reported<br><br>Mean +/- SD in CD:<br>17.8+/-0.85µmol/L<br>Vs HC:<br>26.8+/-0.7µmol/L<br>*P* <0.0001	Not clear / reported	Yes	Intake compared between CD and HC by FFQ estimated macronutrient, fruit and veg. CD intake of energy, protein and Veg lower than HC<br>Vit E in remission vs active *NS*<br>Vit E if BMI <20 vs BMI>20<br>13.4+/-1.5 vs.18.4+/-0.8<br>*P* 0.029	Low

**Vitamin E** Author [main text ref number]	Year	N CD (HC)	P/R	Data on % Deficiency in CD	CD vs HC	Separate reporting of remission? Definition of remission or Cohort characteristics (CCh)	Prevalence of Deficiency In CD remission group or if not separately reported in CD group Difference vs HC	Supports Vit E deficiency being present in CD remission	Supports Vit E status being altered in Cd remission in comparison with healthy controls (HC)	Exploration of cause Subgroup analysis or Special tests	Overall Quality (see table 2)
Fillippi [19]	2006	54 (25)	P	X	?	All in remission<br><br>CDAI<br>All subjects needed 3/12 (CDAI <150)<br><br>Mean CRP 6.6-/-0.8mg/dl	Prevalence (no definition provided)<br>10% (estimated from graph)<br><br>Biochemical analysis of HC not reported (either as prevalence of deficiency or results)	Yes	Not reported	Mean Intake of Vit E assessed with 3-day food diary in CD and compared vs HC in male and female separately<br>Intake in CD f (n=28) vs HC f (n=16) and CD m (n=26) vs HC m (n=9) *NS*<br>Both CD f and HC f mean intake <French RDA (12mg/day)	Low
MacMaster [20]	2021	59		X		All in clinical remission (HBI)<br>No separate CD<br><br>53% in biochem remission (BCR) (FCP < 250, CRP <10 alb >35)<br>(not report separately)	2% deficiency<br><br>3.5 to 9.5 µmol/mmol cholesterol	Yes (low prevalence)	Not reported	Relationship between alb, FCP and crp explored with Spearman's RHO- nil correlation.<br>Exploration of phenotypic predictors of Vit E serum concentrations found no associations<br>Survival analysis of flare in subsequent 12 months found no relationship	Low

Vitamin K

**Vitamin K** Author [main text ref number]	Year	N CD (HC)	P/R	Data on % Deficiency in CD	CD vs HC	Separate reporting of remission? Definition of remission or Cohort characteristics (CCh)	Prevalence of Deficiency In CD remission group or if not separately reported in CD group Difference vs HC	Supports Vitamin K deficiency being present in CD remission	Supports Vitamin K status being altered in Cd remission in comparison with healthy controls (HC)	Exploration of cause Subgroup analysis or Special tests	Overall Quality (see table 2)
Schoon [27]	2001	32 (34)	P		X	All "in remission"- although definition of remission not reported.<br>CCh:<br>Mean (SD) CDAI: 106 (78)	Prevalence not reported<br>Median Vit K 0.42ng/ml Cd<br>Vs<br>0.61 HC *P*<0.01<br><br>Undercarboxylated osteocalcin (Marker of Vitamin K status, elevated in deficiency) higher in CD vs HC	Not reported	Yes	"Free" uncarboxylated osteocalcin lower in CD, and was inversely correlated (and an independent risk factor in multiple linear regression) to lumbar spine bone mineral density (BMD)	Medium

**Vitamin K** Author [main text ref number]	Year	N CD (HC)	P/R	Data on % Deficiency in CD	CD vs HC	Separate reporting of remission? Definition of remission or Cohort characteristics (CCh)	Prevalence of Deficiency In CD remission group or if not separately reported in CD group Difference vs HC	Supports Vitamin K deficiency being present in CD remission	Supports Vitamin K status being altered in Cd remission in comparison with healthy controls (HC)	Exploration of cause Subgroup analysis or Special tests	Overall Quality (see table 2)
Duggan [29]	2004	44 (44)	X	X	X	All in remission- defined as the Absence of GI symptoms	Prevalence not reported<br><br>Undercarboxylated osteocalcin marker of Vitamin K status, elevated in CD 5.1ng/mL vs HC 3.9 ng/mL *P*<0.05	Not reported	Yes	Vitamin K intake estimated by FFQ: Trend to lower intake CD: 117µg vs HC: 147 *P* 0.059 Also measured marker of bore turnover: Urinary N-Telopeptide cross-linked type I collagen as marker of bone turnover- correlated to Undercarboxylated osteocalcin	Low
Nakajima [37]	2011	47 (41)	P		X	No separate remission group<br><br>Cohort characteristics: Mean (+/-SD) CDAI: 107.6+/-88.0	Prevalence not reported<br><br>Undercarboxylated osteocalcin reported as significantly higher in CD than HC (P<0.05) but values only displayed on graph	Not reported	Yes	Undercarboxylated osteocalcin was correlated to CDAI (R=0.36, P<0.0001)<br><br>BMI, CRP not correlated	Low

**Vitamin K** Author [main text ref number]	Year	N CD (HC)	P/R	Data on % Deficiency in CD	CD vs HC	Separate reporting of remission? Definition of remission or Cohort characteristics (CCh)	Prevalence of Deficiency In CD remission group or if not separately reported in CD group Difference vs HC	Supports Vitamin K deficiency being present in CD remission	Supports Vitamin K status being altered in Cd remission in comparison with healthy controls (HC)	Exploration of cause Subgroup analysis or Special tests	Overall Quality (see table 2)
MacMaster [20]	2021	59		X		All in clinical remission (HBI) No separate CD<br><br>53% in biochem remission (BCR) (FCP < 250, CRP <10 alb >35) (not report separately)	2% deficiency<br><br>0.2nmol/mmol triglyceride	No / low prevalence	Yes low	Relationship between alb, FCP and crp explored with Spearman's RHO- nil correlation.<br><br>No exploration of phenotypic predictors of deficiency done.<br><br>Survival analysis of flare in subsequent 12 months found no relationship	Medium

Thiamine (B1)

**Thiamine** Author [main text ref number]	Year	N CD (HC)	P/R	Data on % Deficiency in CD	CD vs HC	Separate reporting of remission? Definition of remission or Cohort characteristics (CCh)	Prevalence of Deficiency In CD remission group or if not separately reported in CD group Difference vs HC	Supports Thiamine deficiency being present in CD remission	Supports Thiamine status being altered in Cd remission in comparison with healthy controls (HC)	Exploration of cause Subgroup analysis or Special tests	Overall Quality (see table 2)
Geerling [16]	1998	32 (32)		X	X	No separate remission group<br><br>(CCh):<br>Median CDAI 139, 53% remission (CDAI <150)	0% deficient (<85nmol/L)<br><br>Mean (+/-SD) CD: 119+/-27.5nmol/L<br>Vs<br>HC: 114+/-26.4<br>*NS*	No	No	Intake of Thiamine assessed with FFQ:<br>Less than RDA in 34% CD and 41% HC. Intake not different<br>No reported intake vs serum analyses.<br>Nil analysis vs disease activity	Medium
Geerling [18]	2000	23 (69)			X	Remission not reported separately (CCh):<br>87% in remission (CDAI <150)<br>Mean (+/-SD) CDAI<br>96.9+/-66.5	No prevalence data.<br><br>Mean+/-SD in CD: 115+/-15.2nmol/L<br>Vs<br>Controls<br>109+/-277nmol/L<br>*NS*	Not reported	No	Intake of Thiamine assessed with FFQ and diary<br>CD vs HC *NS*<br>No reported intake vs serum correlation analyses.	High

**Thiamine** Author [main text ref number]	Year	N CD (HC)	P/R	Data on % Deficiency in CD	CD vs HC	Separate reporting of remission? Definition of remission or Cohort characteristics (CCh)	Prevalence of Deficiency In CD remission group or if not separately reported in CD group Difference vs HC	Supports Thiamine deficiency being present in CD remission	Supports Thiamine status being altered in Cd remission in comparison with healthy controls (HC)	Exploration of cause Subgroup analysis or Special tests	Overall Quality (see table 2)
Filippi [19]	2006	54		X	X	All in remission<br><br>CDAI<br>All subjects needed 3/12 (CDAI <150)<br><br>Mean CRP 6.6-/-0.8mg/dl	32% defic (no reference ranges) in graph only<br><br>Biochemical analysis of HC not reported (either as prevalence of deficiency or results)	Yes	Not reported	Mean Intake of B1 assessed with 3-day food diary in CD and compared vs HC in male and female separately<br>Intake in CD f (n=28) 1.23+/-0.18mg/day less than HC f (n=16) 1.78+/-0.38<br>*P*<0.05<br>CD m (n=26) vs HC m (n=9) *NS*<br>All groups mean > French RDA	Low

**Thiamine** Author [main text ref number]	Year	N CD (HC)	P/R	Data on % Deficiency in CD	CD vs HC	Separate reporting of remission? Definition of remission or Cohort characteristics (CCh)	Prevalence of Deficiency In CD remission group or if not separately reported in CD group Difference vs HC	Supports Thiamine deficiency being present in CD remission	Supports Thiamine status being altered in Cd remission in comparison with healthy controls (HC)	Exploration of cause Subgroup analysis or Special tests	Overall Quality (see table 2)
MacMaster [20]	2021	59		X		All in clinical remission (HBI) No separate CD<br><br>53% in biochem remission (BCR) (FCP < 250, CRP <10 alb >35) (not report separately)	0% deficiency<br><br>275ng/g Hb	No	Not reported	Relationship between alb, FCP and crp explored with Spearman's RHO- nil correlation.<br><br>No exploration of phenotypic predictors of deficiency done.<br><br>Survival analysis of flare in subsequent 12 months found no relationship	Medium

Riboflavin (B2)

**Riboflavin (B2)** Author [main text ref number]	Year	N CD (HC)	P/R	Data on % Deficiency in CD	CD vs HC	Separate reporting of remission? Definition of remission or Cohort characteristics (CCh)	Prevalence of Deficiency In CD remission group or if not separately reported in CD group Difference vs HC	Supports B2 deficiency being present in CD remission	Supports B2 status being altered in Cd remission in comparison with healthy controls (HC)	Exploration of cause Subgroup analysis or Special tests	Overall Quality (see table 2)
MacMaster [20]	2021	59		X		All in clinical remission (HBI) No separate CD<br><br>53% in biochem remission (BCR) (FCP < 250, CRP <10 alb >35) (not report separately)	2% deficient <1.0-3.4 nmol/g Hb	No / low prevalence	Not clear / reported	Relationship between alb, FCP and crp explored with Spearman's RHO. Only Alb and crp related (rho=0.24 p 0.018, rho 0.20 p0.054 respectively) No exploration of phenotypic predictors of deficiency done.<br><br>Survival analysis of flare in subsequent 12 months found no relationship	Medium

Niacin (B3)

**Niacin (B3)** [main text ref number]	Year	N CD (HC)	P/R	Data on % Deficiency in CD	CD vs HC	Separate reporting of remission? Definition of remission or Cohort characteristics (CCh)	Prevalence of Deficiency In CD remission group or if not separately reported in CD group<br><br>Difference vs HC	Supports B3 deficiency being present in CD remission	Supports B3 status being altered in Cd remission in comparison with healthy controls (HC)	Exploration of cause Subgroup analysis or Special tests	Overall Quality (see table 2)
Filippi [19]	2006	54		X	X	All in remission<br><br>CDAI<br>All subjects needed 3/12 (CDAI <150)<br><br>Mean CRP 6.6-/-0.8mg/dl	77% of CD low in Niacin (no ref range)<br><br>Biochemical analysis of HC not reported (either as prevalence of deficiency or results)	Yes	Not clear / reported	Mean B3 intake assessed with 3-day food diary in CD and compared vs HC in male and female separately Intake in CD f (n=28) vs HC f (n=16) and CD m (n=26) vs HC m (n=9) *NS*<br>All groups > French RDA.	Low

Pyridoxic Acid (B6)

**Pyridoxic Acid (B6)** Author [main text ref number]	Year	N CD (HC)	P/R	Data on % Deficiency in CD	CD vs HC	Separate reporting of remission? Definition of remission or Cohort characteristics (CCh)	Prevalence of Deficiency In CD remission group or if not separately reported in CD group Difference vs HC	Supports B6 deficiency being present in CD remission	Supports B6 status being altered in Cd remission in comparison with healthy controls (HC)	Exploration of cause Subgroup analysis or Special tests	Overall Quality (see table 2)
Filippi [19]	2006	54		X	X	All in remission<br><br>CDAI<br>All subjects needed 3/12 (CDAI <150)<br><br>Mean CRP 6.6-/-0.8mg/dl	34%<br>(no reference ranges) in graph only<br><br>Biochemical analysis of HC not reported (either as prevalence of deficiency or results)	Yes	Not clear / reported	Mean Intake of B6 assessed with 3-day food diary in CD and compared vs HC in male and female separately<br>Intake in CD f (n=28) 1.04+/-0.0.7mg/day less than HC f (n=16) 1.57+/-0.20 *P*<0.01<br>CD m (n=26) vs HC m (n=9)<br>*NS*<br>CD F mean, CD M and HC M < French RDA	Low

**Pyridoxic Acid (B6)** Author [main text ref number]	Year	N CD (HC)	P/R	Data on % Deficiency in CD	CD vs HC	Separate reporting of remission? Definition of remission or Cohort characteristics (CCh)	Prevalence of Deficiency In CD remission group or if not separately reported in CD group Difference vs HC	Supports B6 deficiency being present in CD remission	Supports B6 status being altered in Cd remission in comparison with healthy controls (HC)	Exploration of cause Subgroup analysis or Special tests	Overall Quality (see table 2)
Vagianos [34]	2007	84		X		No separate remission CD group<br><br>CCh:<br>61% in remission (HBI<5)	45.7% (<5μg/L)	Yes	Not clear / reported	Intake assessed with FFQ then 4-day food diary inc supplements then compared vs adequacy (defined as <66% dietary reference value (DRV)) B6 intake inadequate in 4.2% CD<br><br>Spearman Correlation of B6 Intake / 100kcal vs serum B6 very strongly correlated *r* 0.72 P<0.001	Medium

**Pyridoxic Acid (B6)** Author [main text ref number]	Year	N CD (HC)	P/R	Data on % Deficiency in CD	CD vs HC	Separate reporting of remission? Definition of remission or Cohort characteristics (CCh)	Prevalence of Deficiency In CD remission group or if not separately reported in CD group Difference vs HC	Supports B6 deficiency being present in CD remission	Supports B6 status being altered in Cd remission in comparison with healthy controls (HC)	Exploration of cause Subgroup analysis or Special tests	Overall Quality (see table 2)
Vagianos [39]	2012	70	P	X		No separate remission CD group<br><br>CCh:<br>62.9% in remission (HBI<5) at every visit.	30% (<20nmol/L)	Yes	Not clear / reported	Intake assessed with FFQ then 3-day food diary inc. supplements (57.1% on supplement)<br><br>Pearson Correlation of B6 Intake vs serum B6 strongly correlated *r* 0.41 *P*<0.001<br><br>59% of B6 deficient subjects had previous bowel surgery vs 13% without. B6 not correlated with HcY	High

**Pyridoxic Acid (B6)** Author [main text ref number]	Year	N CD (HC)	P/R	Data on % Deficiency in CD	CD vs HC	Separate reporting of remission? Definition of remission or Cohort characteristics (CCh)	Prevalence of Deficiency In CD remission group or if not separately reported in CD group Difference vs HC	Supports B6 deficiency being present in CD remission	Supports B6 status being altered in Cd remission in comparison with healthy controls (HC)	Exploration of cause Subgroup analysis or Special tests	Overall Quality (see table 2)
MacMaster [20]	2021	59	P	X		All in clinical remission<br><br>53% in biochem remission (BCR) (FCP < 250, CRP <10 Alb >35) (not report separately)	10%<br>250 pmol/g Hb	Yes	Yes	Relationship between Alb, FCP and crp explored with Spearman's RHO- nil correlation.<br><br>Exploration of phenotypic predictors of deficiency found B6 deficiency to be more common in structuring than inflammatory phenotype P=0.001<br><br>Survival analysis of flare in subsequent 12 months found no relationship	Medium

Folate (B9)

**Folate (B9)** Author [main text ref number]	Year	N CD (HC)	P/R	Data on % Deficiency in CD	CD vs HC	Separate reporting of remission? Definition of remission or Cohort characteristics (CCh)	Prevalence of Deficiency In CD remission group or if not separately reported in CD group<br><br>Difference vs HC	Supports Folate deficiency being present in CD remission	Supports Folate status being altered in Cd remission in comparison with healthy controls (HC)	Exploration of cause Subgroup analysis or Special tests	Overall Quality (see table 2)
Imes [23]	1987	137	P	X		Remission not reported separately<br><br>CCr:<br>70% in remission (CDAI <150)	10% (no reference range)	Yes	Not clear / reported	nil	Low
Geerling [16]	1998	32	P	X		No separate remission group<br><br>(CCh):<br>Median CDAI 139,<br>53% remission (CDAI <150)	No reporting of prevalence of deficiency<br>Mean +/-SD<br>CD: 14.4+/-13.4<br>vs<br>HC: 13.4+/-5.88<br>*NS*	Not clear / reported	No	Intake not among micronutrients assessed	Medium
Geerling [18]	2000	23	P		X	Remission not reported separately<br>(CCh):<br>87% in remission (CDAI <150)<br>Mean (+/-SD) CDAI<br>96.9+/-66.5	No reporting of prevalence of deficiency<br><br>Mean +/-SD<br>CD:10.7+/-9.1<br>Vs<br>HC: 12.4+/-5.6<br>*NS*	Not clear / reported	No	Intake not among micronutrients assessed	Medium

**Folate (B9)** Author [main text ref number]	Year	N CD (HC)	P/R	Data on % Deficiency in CD	CD vs HC	Separate reporting of remission? Definition of remission or Cohort characteristics (CCh)	Prevalence of Deficiency In CD remission group or if not separately reported in CD group<br><br>Difference vs HC	Supports Folate deficiency being present in CD remission	Supports Folate status being altered in Cd remission in comparison with healthy controls (HC)	Exploration of cause Subgroup analysis or Special tests	Overall Quality (see table 2)
Koutrobakis [25]	2000	55 (74)	?		X	No separate remission group.<br><br>CCh: 69% in remission (CDAI<150)	Mean +/-SD CD: 6.34+/-3.05ng/ml vs HC: 8.78 +/-3.07ng/ml *P* <0.05	Not clear / reported	Yes	Negatively correlated with homocysteine folate levels (r=-0.493, P < 0.01)	Low
Filippi [19]	2006	54			X	All in remission<br><br>CDAI All subjects needed 3/12 (CDAI <150)<br><br>Mean CRP 6.6-/-0.8mg/dl (~63nmol/L)	20-25% (no reference ranges) in graph only<br><br>Biochemical analysis of HC not reported (either as prevalence of deficiency or results)	Yes	Not clear / reported	Mean B9 intake assessed with 3-day food diary in CD and compared vs HC in male and female separately Intake in CD f (n=28) vs HC f (n=16) and CD m (n=26) vs HC m (n=9) *NS* All groups >French RDA.	Low

**Folate (B9)** Author [main text ref number]	Year	N CD (HC)	P/R	Data on % Deficiency in CD	CD vs HC	Separate reporting of remission? Definition of remission or Cohort characteristics (CCh)	Prevalence of Deficiency In CD remission group or if not separately reported in CD group<br><br>Difference vs HC	Supports Folate deficiency being present in CD remission	Supports Folate status being altered in Cd remission in comparison with healthy controls (HC)	Exploration of cause Subgroup analysis or Special tests	Overall Quality (see table 2)
Roblin [33]	2007	92	P	X		No separate remission group.<br><br>Cohort characteristics 38/55 (69%) in CDAI remission	35% (RBC folate <210nmol/L)<br><br>No HC comparators	Yes	Not clear / reported	Primary aim of study was exploration of Hyperhomocysteinaemia (HHcy) against BMD.<br><br>Low level folate assoc with HHcy and low BMD	Medium
Vagianos [34]	2007	84		X		No separate remission CD group<br><br>CCh:<br>61% in remission (HBI<5)	0%<br><br>(Red blood cell folate <320μg/L)	No	Not clear / reported	Intake assessed with FFQ then 4-day food diary inc supplements then compared vs adequacy (defined as <66% dietary reference value (DRV))<br>B9 intake inadequate in 20% CD Spearman Correlation of B9 Intake / 100kcal vs serum B9 strongly correlated *r* 0.57 *P*<0.0001	Medium
Valentini [35]	2008	94	P	X	X	All in remission (CDAI <150)	0% (<2.8μg/L)<br><br>Conc vs sex matched HC *NS*	No	No	nil	High

**Folate (B9)**<br>Author<br>[main text ref number]	Year	N CD (HC)	P/R	Data on % Deficiency in CD	CD vs HC	Separate reporting of remission?<br>Definition of remission<br>or Cohort characteristics (CCh)	Prevalence of Deficiency<br>In CD remission group or if not separately reported in CD group<br><br>Difference vs HC	Supports Folate deficiency being present in CD remission	Supports Folate status being altered in Cd remission in comparison with healthy controls (HC)	Exploration of cause Subgroup analysis or Special tests	Overall Quality (see table 2)
Kallel [36]	2011	89 (103)	P		X	Remission CD not reported separately<br><br>CCh: 71.9% in remission (CDAI<150)	Mean +/-SD<br>CD: 8.54+/-3.04<br>vs<br>HC: 8.10+/-3.11<br>*NS*	Not reported	No	In CD cases Vit B9 was identified as having a weak correlation with Homocysteine (HcY)<br><br>*R*=-0.22 *P*=0.03	High
Vagianos [39]	2012	70	P	X		No separate remission CD group<br><br>CCh:<br>62.9% in remission (HBI<5)<br>at every visit.	1.4%<br>(<11.8nmol/L)<br><br>((64.3% on supplements))	No / low prevalence	Not clear / reported	Intake assessed with FFQ then 3-day food diary inc supplements (64.3% on supplement)<br><br>Pearson Correlation of B9 Intake vs serum weakly correlated<br>*r* 0.26 *P*0.008<br><br>B9 not correlated with HcY	High

**Folate (B9)** Author [main text ref number]	Year	N CD (HC)	P/R	Data on % Deficiency in CD	CD vs HC	Separate reporting of remission? Definition of remission or Cohort characteristics (CCh)	Prevalence of Deficiency In CD remission group or if not separately reported in CD group<br><br>Difference vs HC	Supports Folate deficiency being present in CD remission	Supports Folate status being altered in Cd remission in comparison with healthy controls (HC)	Exploration of cause Subgroup analysis or Special tests	Overall Quality (see table 2)
Bermejo [40]	2013	180	P	X		62% in remission (HBI<5)	22.2% (<3ng/ml)	Yes	Not reported	multivariate analysis of potential risk factors identified HBI>2 as a risk factor (OR 2.4 (1.2-5.1) *P* 0.01), whereas CRP<10, ESR>20, anaemia, age, sex, disease location and ileal resections not related	Medium
Lupu [45]	2015	113	P	X		Remission not reported separately<br><br>CCh:<br>62% remission (HBI<5)	5.2% (<4.6ng/ml)	Yes	Not reported	nil	Low

**Folate (B9)** Author [main text ref number]	Year	N CD (HC)	P/R	Data on % Deficiency in CD	CD vs HC	Separate reporting of remission? Definition of remission or Cohort characteristics (CCh)	Prevalence of Deficiency In CD remission group or if not separately reported in CD group<br><br>Difference vs HC	Supports Folate deficiency being present in CD remission	Supports Folate status being altered in Cd remission in comparison with healthy controls (HC)	Exploration of cause Subgroup analysis or Special tests	Overall Quality (see table 2)
De Castro [53]	2019	31	P	X		Yes. Remission defined with endoscopy (<5, no deep ulcers or) or MRI with none or creeping fat, deep ulcers or oedema.<br><br>Mean (SD) CDAI of this group 41.35(+/-41.2)	0% (3.9ng/mL)	No	Not reported	Folate lower in those with endoscopic or MRI activity 9.82(+/-3.8) vs 12.7 (+/-4.09) *P* 0.0012 than those without	Medium
Marcil [54]	2019	274	P	X		Remission not reported separately<br><br>Cohort characteristics: 58% remission (HBI<5)	3.3% (<7nmol/ml) or (<317nmol/L RBC)	No (low prevalence)	Not reported	nil	Low

**Folate (B9)** Author [main text ref number]	Year	N CD (HC)	P/R	Data on % Deficiency in CD	CD vs HC	Separate reporting of remission? Definition of remission or Cohort characteristics (CCh)	Prevalence of Deficiency In CD remission group or if not separately reported in CD group<br><br>Difference vs HC	Supports Folate deficiency being present in CD remission	Supports Folate status being altered in Cd remission in comparison with healthy controls (HC)	Exploration of cause Subgroup analysis or Special tests	Overall Quality (see table 2)
MacMaster [20]	2021	59	P	X		All in clinical remission<br><br>53% in biochem remission (BCR) (FCP < 250, CRP <10 alb >35) (not report separately)	9%<br><br>(3ng/mL)	Yes	Not clear / reported	Relationship between alb, FCP and crp explored with Spearman's RHO- nil correlation.<br><br>Exploration of phenotypic predictors of deficiency including supplements (n=7) found no associations<br><br>Survival analysis of flare in subsequent 12 months found no relationship	Medium

Cobalamin (B12)

**Cobalamin (B12)** Author [main text ref number]	Year	N CD (HC)	P/R	Data on % Deficiency in CD	CD vs HC	Separate reporting of remission? Definition of remission or Cohort characteristics (CCh)	Prevalence of Deficiency In CD remission group or if not separately reported in CD group Difference vs HC	Supports B12 deficiency being present in CD remission	Supports B12 status being altered in Cd remission in comparison with healthy controls (HC)	Exploration of cause Subgroup analysis or Special tests	Overall Quality (see table 2)
Imes [23]	1987	137	P	X		Remission not reported separately<br><br>CCr:<br>70% in remission (CDAI <150)	4% (no reference range)	Yes	Not clear / reported	nil	Low
Geerling [16]	1998	32	P		X	No separate remission group<br><br>(CCh):<br>Median CDAI 139,<br>53% remission (CDAI <150)	Mean +/-SD<br>CD: 403+/-282<br>Vs<br>HC: 263+/-91.5<br>*NS*	Not clear / reported	No	Intake not among micronutrients assessed	Medium

**Cobalamin (B12)** Author [main text ref number]	Year	N CD (HC)	P/R	Data on % Deficiency in CD	CD vs HC	Separate reporting of remission? Definition of remission or Cohort characteristics (CCh)	Prevalence of Deficiency In CD remission group or if not separately reported in CD group Difference vs HC	Supports B12 deficiency being present in CD remission	Supports B12 status being altered in Cd remission in comparison with healthy controls (HC)	Exploration of cause Subgroup analysis or Special tests	Overall Quality (see table 2)
Geerling [18]	2000	23	P		X	Remission not reported separately (CCh): 87% in remission (CDAI <150) Mean (+/-SD) CDAI 96.9+/-66.5	Mean +/-SD CD: 225+/-60.7 Vs HC: 270+/-88.2 *P* <0.05	Not clear / reported	Yes	Intake not among micronutrients assessed	Medium
Koutrobakis [25]	2000	55	?		X	No separate remission group. CCh: 69% in remission (CDAI<150)	Mean +/-SD (pg/ml) CD: 666.5+/-366.8 vs HC: 377.5+/-155.6 *P* <0.05	Not reported	No, it shows that B12 higher in CD than HC	Not correlated with Homocysteine	Low
Tajika [30]	2004	33 (15)			X	No separate reporting of remission. CCh: Mean CDAI 84.1 +/-44.2 (range, 12.6–182.7)	Mean (SD) B12 CD: 479.1+/-302.7 vs HC 486.0 +/-90.1 *NS*	Not reported	No	Nil relationship to 25 OD in correlation (study was primarily reported as a 25 OH D paper)	High

**Cobalamin (B12)**<br>Author [main text ref number]	Year	N CD (HC)	P/R	Data on % Deficiency in CD	CD vs HC	Separate reporting of remission? Definition of remission or Cohort characteristics (CCh)	Prevalence of Deficiency<br>In CD remission group or if not separately reported in CD group<br>Difference vs HC	Supports B12 deficiency being present in CD remission	Supports B12 status being altered in Cd remission in comparison with healthy controls (HC)	Exploration of cause Subgroup analysis or Special tests	Overall Quality (see table 2)
Filippi [19]	2006	54		X	X	All in remission (CDAI <150)<br>All in remission<br><br>CDAI<br>All subjects needed 3/12 (CDAI <150)<br><br>Mean CRP 6.6-/-0.8mg/dl (~63nmol/L)	30-35%<br>(no reference ranges)<br>Graph only<br><br>Biochemical analysis of HC not reported (either as prevalence of deficiency or results)	Yes	Not clear / reported	Mean B12 intake assessed with 3-day food diary in CD and compared vs HC in male and female separately<br><br>Intake in CD f (n=28) vs HC f (n=16) and CD m (n=26) vs HC m (n=9)<br>*NS*<br>All groups > French RDA.	Low
Roblin [33]	2007	92	P	X		No separate remission group.<br><br>Cohort characteristics 38/55 (69%) in CDAI remission	23%<br>(<145pmol/L)<br><br>No HC comparators	Yes	Not clear / reported	Primary aim of study was exploration of Hyperhomocysteinaemia (HHcy) against BMD.<br><br>Low level B12 not assoc with HHcy	Medium

**Cobalamin (B12)** Author [main text ref number]	Year	N CD (HC)	P/R	Data on % Deficiency in CD	CD vs HC	Separate reporting of remission? Definition of remission or Cohort characteristics (CCh)	Prevalence of Deficiency In CD remission group or if not separately reported in CD group Difference vs HC	Supports B12 deficiency being present in CD remission	Supports B12 status being altered in Cd remission in comparison with healthy controls (HC)	Exploration of cause Subgroup analysis or Special tests	Overall Quality (see table 2)
Vagianos [34]	2007	84		X		No separate remission CD group<br><br>CCh:<br>61% in remission (HBI<5)	24.1%<br><br>(<179pmol/L)	No	Not clear / reported	Intake assessed with FFQ then 4-day food diary inc. supplements then compared vs adequacy (defined as <66% dietary reference value (DRV))<br>B12 intake inadequate in 5.6% CD Spearman Correlation of B12 Intake / 100kcal vs serum B12 moderately correlated *r* 0.45 *P* 0.0001	Medium
Valentini [35]	2008	94	P	X	X	All in remission (CDAI <150)	9.6% (<199ng/L)<br><br>Conc vs sex matched HC *NS*	Yes	No	nil	High
Kallel [36]	2011	89	P		X	Remission CD not reported separately<br><br>CCh: 71.9% in remission (CDAI<150)	Mean +/-SD<br>CD: 295+/-160 ng/l<br>Vs<br>HC:378+/-170 ng/l<br>*P*<0.001	Not reported	Yes	In CD cases Vit B12 was identified as having a strong negative correlation with Homocysteine (HcY)<br><br>*R*=-0.43 *P*<0.001	High

**Cobalamin (B12)** Author [main text ref number]	Year	N CD (HC)	P/R	Data on % Deficiency in CD	CD vs HC	Separate reporting of remission? Definition of remission or Cohort characteristics (CCh)	Prevalence of Deficiency In CD remission group or if not separately reported in CD group Difference vs HC	Supports B12 deficiency being present in CD remission	Supports B12 status being altered in Cd remission in comparison with healthy controls (HC)	Exploration of cause Subgroup analysis or Special tests	Overall Quality (see table 2)
Vagianos [39]	2012	70	P	X		No separate remission CD group<br><br>CCh:<br>62.9% in remission (HBI<5) at every visit.	11.4% (<180pmol/L)	Yes	Not clear / reported	Intake assessed with FFQ then 3-day food diary inc supplements (47.1% on oral supplement, 4.3% on IM)<br><br>Pearson Correlation of B12 Intake vs serum strongly correlated *r* 0.42 *P*<0.001<br><br>B12 weakly negatively correlated with HcY r-0.241 *P* 0.008	High
Bermejo [40]	2013	180	P	X		62% in remission (HBI<5)	15.6% (<200 pg/ml)	Yes	Not reported	multivariate analysis of potential risk factors only identified ileal resection as a risk factor (OR 2.7 (1.2-6.7) *P* 0.02), whereas CRP<10, ESR>20, anaemia, age, sex, disease location and HBI>2 not related	Medium

**Cobalamin (B12)** Author [main text ref number]	Year	N CD (HC)	P/R	Data on % Deficiency in CD	CD vs HC	Separate reporting of remission? Definition of remission or Cohort characteristics (CCh)	Prevalence of Deficiency In CD remission group or if not separately reported in CD group Difference vs HC	Supports B12 deficiency being present in CD remission	Supports B12 status being altered in Cd remission in comparison with healthy controls (HC)	Exploration of cause Subgroup analysis or Special tests	Overall Quality (see table 2)
Lupu [45]	2015	115	P	X		Remission not reported separately<br><br>CCh:<br>CDAI Median (IQR):<br>96 (51-145)	13.0%<br>(<191pg/ml)	Yes	Not clear / reported	Nil	Low
Ward [47]	2015	381	R	X		Remission group reported separately (HBI<5)	33% deficient<br><br>(Holotranscobalamin <25nmol, or 25-50nmol/L with Methylmalonic >280 nM/L, comparative low B12 <147pmol/l)	Yes	Not reported	Univariate analysis of risk factors for B12 deficiency identified:<br>Resection <20cm:<br>OR 3.0 (95% CI 1.5-6.0)<br>*P* 0.002<br>Resection >20cm:<br>OR 6.7 (95% 3.0-14.7)<br>*P* 0.0001<br>TI inflammation OR:<br>OR 3.9 (95% CI 2.2-6.9)<br>as leading predictors	High

**Cobalamin (B12)** Author [main text ref number]	Year	N CD (HC)	P/R	Data on % Deficiency in CD	CD vs HC	Separate reporting of remission? Definition of remission or Cohort characteristics (CCh)	Prevalence of Deficiency In CD remission group or if not separately reported in CD group Difference vs HC	Supports B12 deficiency being present in CD remission	Supports B12 status being altered in Cd remission in comparison with healthy controls (HC)	Exploration of cause Subgroup analysis or Special tests	Overall Quality (see table 2)
Battat [49]	2017	66	P	X		Remission not reported separately<br><br>CCh:<br>83.3% Remission (HBI<5)	7.6%<br>(B12 <148pmol/L)<br><br>3%<br>B12 <148pmol/L and MMA >280nM/L)<br><br>6%<br>4 isolated MMA elevation<br>(>280nMol/L)	Yes	Not reported	Mean B12 if Ileal resection >30cm (n=5) lower those than those with either no resection or <30cm resected (n=60).<br>177.0 vs 259.9 *P* 0.02<br><br>FUT2 mutations explored vs B12 and deficiency. No effect on percentage deficient, but homozygous FUT2 mutations had higher B12	Medium

**Cobalamin (B12)** Author [main text ref number]	Year	N CD (HC)	P/R	Data on % Deficiency in CD	CD vs HC	Separate reporting of remission? Definition of remission or Cohort characteristics (CCh)	Prevalence of Deficiency In CD remission group or if not separately reported in CD group Difference vs HC	Supports B12 deficiency being present in CD remission	Supports B12 status being altered in Cd remission in comparison with healthy controls (HC)	Exploration of cause Subgroup analysis or Special tests	Overall Quality (see table 2)
De Castro [53]	2019	31	P	X		Yes. Remission defined with endoscopy (<5, no deep ulcers or) or MRI with none or creeping fat, deep ulcers or oedema.<br><br>Mean (SD) CDAI of this group 41.35(+/-41.2)	4.17% (<197pg/mL)	No	Not reported	Mean B12 compared in remission group to those with endoscopy / MRI activity- *NS*	Medium
Marcil [54]	2019	274	P	X		Remission CD not reported separately<br><br>58% remission (HBI<5)	26.3%<br><br>(<148pmol/L, or on prescribed supplement)	Yes	Not clear / reported	52.7% of those with low B12 were on supplements	Low

**Cobalamin (B12)** Author [main text ref number]	Year	N CD (HC)	P/R	Data on % Deficiency in CD	CD vs HC	Separate reporting of remission? Definition of remission or Cohort characteristics (CCh)	Prevalence of Deficiency In CD remission group or if not separately reported in CD group Difference vs HC	Supports B12 deficiency being present in CD remission	Supports B12 status being altered in Cd remission in comparison with healthy controls (HC)	Exploration of cause Subgroup analysis or Special tests	Overall Quality (see table 2)
McMaster [20]	2020	59	P	graph		All in clinical remission<br><br>53% in biochem remission (BCR) (FCP < 250, CRP <10 Alb >35) (not report separately)	12% (estimated from graph)	Yes	Not clear / reported	Relationship between Alb, FCP and crp explored with Spearman's RHO- nil correlation.<br><br>Exploration of phenotypic predictors of deficiency including supplements (n=4) found no associations<br><br>Survival analysis of flare in subsequent 12 months found no relationship	Low

Homocysteine (HcY)

**Homocysteine (HcY)** [main text ref number]	Year	N CD (HC)	P/R	Data on % Deficiency in CD	CD vs HC	Separate reporting of remission? method used or % remission in group	Prevalence of Hyperhomocysteinaemia (HHcy) In CD remission group or if not separately reported in CD group<br><br>Difference vs HC	Supports hyperHcY being present in CD remission	Supports HcY status being elevated in CD remission in comparison with healthy controls (HC)	Exploration of cause Subgroup analysis or Special tests	Overall Quality (see table 2)
Koutrobakis [25]	2000	55			X	No separate remission group.<br><br>CCh: 69% in remission (CDAI<150)	Mean +/-SD (µmol/L)<br>CD: 13.62+/-6.55<br>vs<br>HC: 9.59+/-3.39<br>P <0.05	Not reported	Yes	homocysteine Negatively correlated with folate levels<br>(r=-0.493,<br>P < 0.01)	Low
Roblin [33]	2007	92	P	X		No separate remission group.<br><br>Cohort characteristics 38/55 (69%) in CDAI remission	60% (>15µmol/l)<br><br>No HC comparators	Yes	Not reported	Primary aim of study was exploration of HHcy against BMD.<br><br>Univariate analysis found HHcy assoc with low osteoporosis (OR: 3.6 95% CI 3.98-250)	Medium

**Homocysteine (HcY)** [main text ref number]	Year	N CD (HC)	P/R	Data on % Deficiency in CD	CD vs HC	Separate reporting of remission? method used or % remission in group	Prevalence of Hyperhomocysteinaemia (HHcy) In CD remission group or if not separately reported in CD group<br><br>Difference vs HC	Supports hyperHcY being present in CD remission	Supports HcY status being elevated in CD remission in comparison with healthy controls (HC)	Exploration of cause Subgroup analysis or Special tests	Overall Quality (see table 2)
Kallel [36]	2011	89	P	X	X	Remission CD not reported separately<br><br>CCh: 71.9% in remission (CDAI<150)	31.5% (>15µmol/l)<br><br>Mean (SD)<br>CD: 13.7+/-4.84<br>vs<br>HC<br>10.8+/-2.80<br>*P*<0.001	Yes	Yes	multivariate analysis showed that HHC was positively associated with age [multi-adjusted odds-ratio (95% confidence interval): 1.14 (1.06–1.24); p<0.001], active disease [7.54 (1.15–49.3); p=0.03], disease duration [8.69 (1.53–49.3); p=0.02] and inversely related to plasma folate [0.64 (0.48–0.84); p=0.002] and vitamin B12 (0.993 (0.987–0.999); p=0.02]	High
Vagianos [39]	2012	70	P	X		No separate remission CD group<br><br>CCh:<br>62.9% in remission (HBI<5) at every visit.	No data on baseline % with HyperHcY<br><br>15.7% fluctuating- i.e., 1 abnormal<br><br>2.9% persistent (>13µmol/L)	Yes	Not reported	Serum B12 weakly negatively correlated with HcY<br>r-0.241 *P* 0.008	High

Vitamin C

**Vitamin C** Author [main text ref number]	Year	N CD (HC)	P/R	Data on % Deficiency in CD	CD vs HC	Separate reporting of remission? Definition of remission or Cohort characteristics (CCh)	Prevalence of Deficiency In CD remission group or if not separately reported in CD group<br><br>Difference vs HC	Supports Vitamin C deficiency being present in CD remission	Supports Vitamin C status being altered in Cd remission in comparison with healthy controls (HC)	Exploration of cause Subgroup analysis or Special tests	Overall Quality (see table 2)
Imes [22]	1986	137	P	X		Remission not reported separately<br><br>CCh:<br>70% in remission (CDAI <150)	15% deficient serum ascorbate <30µmol/L<br><br>37% low by leucocyte ascorbate <0.7µmol/10^9 cells	Yes	Not clear / reported	Intake of supplement and diet was below RDA in 20%. No diet vs serum analysis reported<br><br>Serum or leucocyte ascorbate not correlated to CDAI	Medium
Geerling [16]	1998	32	P	X	X	No separate remission group<br><br>(CCh):<br>Median CDAI 139, 53% remission (CDAI <150)	Prevalence of deficiency (<11µmol/L): 50% (from graph)<br><br>Mean +/-SD<br>CD: 29.2+/-10.7 µmol/l<br>Vs<br>HC: 34.8+/-8.6 in (*P* <0.05)	Yes	Yes	Intake assessed with FFQ:<br>Less than RDA 53% CD and 34% HC. Intake NS different<br><br>No reported intake vs serum correlation analyses.	Medium

**Vitamin C** Author [main text ref number]	Year	N CD (HC)	P/R	Data on % Deficiency in CD	CD vs HC	Separate reporting of remission? Definition of remission or Cohort characteristics (CCh)	Prevalence of Deficiency In CD remission group or if not separately reported in CD group<br><br>Difference vs HC	Supports Vitamin C deficiency being present in CD remission	Supports Vitamin C status being altered in Cd remission in comparison with healthy controls (HC)	Exploration of cause Subgroup analysis or Special tests	Overall Quality (see table 2)
Geerling [17]	1999	62	P		X	Yes, mean of 50 Cd remission reported<br><br>(defined by Van Hees <150)	Prevalence of deficiency not reported<br><br>Median (IQR) in CD remission: 38.0 (26.8-57.0) µmol/l<br>Vs<br>HC: 31.0 (27.0-38.3) µmol/l<br>*P* 0.02	Not clear / reported	Yes	Serum Vit C in CDa vs CDr *NS*	Medium
Geerling [18]	2000	23	P		X	Remission not reported separately (CCh):<br>87% in remission (CDAI <150)<br>Mean (+/-SD) CDAI 96.9+/-66.5	Prevalence of deficiency not reported<br><br>Mean +/- SD in CD:<br>54.5+/-22.9 µmol/L<br>Vs HC:<br>47.6+/17.7µmol/L<br>*NS*	Not clear / reported	No	Intake of Vit C<br><br>assessed with FFQ and diary<br>CD vs HC *NS*<br>No reported intake vs serum correlation analyses.	High

**Vitamin C** Author [main text ref number]	Year	N CD (HC)	P/R	Data on % Deficiency in CD	CD vs HC	Separate reporting of remission? Definition of remission or Cohort characteristics (CCh)	Prevalence of Deficiency In CD remission group or if not separately reported in CD group<br><br>Difference vs HC	Supports Vitamin C deficiency being present in CD remission	Supports Vitamin C status being altered in Cd remission in comparison with healthy controls (HC)	Exploration of cause Subgroup analysis or Special tests	Overall Quality (see table 2)
Wendland [28]	2000	34	P		X	Not for comparison of Vit A with HC<br>CCh:<br>70% in remission (CDAI <150)<br>CDAI Mean +/- SEM 141+/-18.66	Vitamin C (mean +/-SEM) lower in CD:<br>64.03+/-4.6 than HC:78.4+/-2.9<br>*P*<0.05<br><br>**mean +/- SEM in CDr (CDAI<150) 69.8+/-6.3	Not reported	No	Intake of Vit C in CD not reduced vs HC on 7-day food diary. No correlation analyses of intake to plasma concentration<br>Vit C concentration negatively correlated to CDAI, serum osromucoid, but not lipid peroxidation as measured by breath pentanes (which was lower in CD vs HC)	Medium

**Vitamin C** Author [main text ref number]	Year	N CD (HC)	P/R	Data on % Deficiency in CD	CD vs HC	Separate reporting of remission? Definition of remission or Cohort characteristics (CCh)	Prevalence of Deficiency In CD remission group or if not separately reported in CD group<br><br>Difference vs HC	Supports Vitamin C deficiency being present in CD remission	Supports Vitamin C status being altered in Cd remission in comparison with healthy controls (HC)	Exploration of cause Subgroup analysis or Special tests	Overall Quality (see table 2)
Filippi [19]	2006	54	P	X	X	All in remission (CDAI <150) All in remission<br><br>CDAI<br>All subjects needed 3/12 (CDAI <150)<br><br>Mean CRP 6.6-/-0.8mg/dl	85%<br>(no reference ranges)<br>in graph only<br><br>Biochemical analysis of HC not reported (either as prevalence of deficiency or results)	Yes	Not clear / reported	Mean Vit C intake assessed with 3-day food diary in CD and compared vs HC in male and female separately<br>Intake CD F < HC F 46.07+/-5.62 vs 96.55+/-12.84 *P*<0.005<br>And CD M < HC M: 56.10+/-8.43 vs 96.75+/-15.76 P<0.05<br>All groups < French RDA	Low

**Vitamin C** Author [main text ref number]	Year	N CD (HC)	P/R	Data on % Deficiency in CD	CD vs HC	Separate reporting of remission? Definition of remission or Cohort characteristics (CCh)	Prevalence of Deficiency In CD remission group or if not separately reported in CD group<br><br>Difference vs HC	Supports Vitamin C deficiency being present in CD remission	Supports Vitamin C status being altered in Cd remission in comparison with healthy controls (HC)	Exploration of cause Subgroup analysis or Special tests	Overall Quality (see table 2)
MacMaster [20]	2021	59	P	X		All in clinical remission<br><br>53% in biochem remission (BCR) (FCP < 250, CRP <10 alb >35) (not report separately)	17% (<15µmol/L)	Yes	Not clear / reported	Relationship between alb, FCP and crp explored with Spearman's RHO- nil correlation.<br><br>Exploration of phenotypic predictors of Vit C serum concentrations found no associations<br><br>Survival analysis vs flare in subsequent 12 months found no relationship	Medium

Calcium

**Calcium** Author [main text ref number]	Year	N CD (HC)	P/R	Data on % Deficiency in CD	CD vs HC	Separate reporting of remission? method used or % remission in group	Prevalence of Deficiency In CD remission group or if not separately reported in CD group<br><br>Difference vs HC	Supports Calcium deficiency being present in CD remission	Supports Calcium status being altered in Cd remission in comparison with healthy controls (HC)	Exploration of cause Subgroup analysis or Special tests	Overall Quality (see table 2)
Tajika [30]	2004	33 (15)			X	No separate reporting of remission. CCh: Mean CDAI 84.1 +/-44.2 (range, 12.6–182.7)	No reported prevalence<br><br>Mean (SD) Calcium CD: 9.3+/-0.7 vs HC 9.2 +/-0.3<br>*NS*	Not reported	No	Nil relationship to 25 OD in correlation (study was primarily reported as a 25 OH D paper)	High
Fillipi [19]	2006	54	P	X	X	All in remission (CDAI <150) All in remission<br><br>CDAI<br>All subjects needed 3/12 (CDAI <150)<br><br>Mean CRP 6.6-/-0.8mg/dl (~63nmol/L)	5% (estimated from graph) (no reference ranges)<br><br>Biochemical analysis of HC not reported (either as prevalence of deficiency or results)	Yes	Not reported	Mean Ca intake assessed with 3-day food diary in CD and compared vs HC in male and female separately<br><br>Intake in CD f (n=28) vs HC f (n=16) and CD m (n=26) vs HC m (n=9)<br>*NS*<br>All groups < French RDA (900mg).	Low

Copper

**Copper** Author [main text ref number]	Year	N CD (HC)	P/R	Data on % Deficiency in CD	CD vs HC	Separate reporting of remission? method used or % remission in group	Prevalence of Deficiency In CD remission group or if not separately reported in CD group Difference vs HC	Supports Copper deficiency being present in CD remission	Supports Copper status being altered in Cd remission in comparison with healthy controls (HC)	Exploration of cause Subgroup analysis or Special tests	Overall Quality (see table 2)
Geerling [16]	1998	32	P	X	X	No separate remission group<br><br>(CCh): Median CDAI 139, 53% remission (CDAI <150)	No deficient cases (<12µmol/L):<br>Mean +/-SD<br>CD: 19.1 +/-4.61 µmol/l<br>Vs<br>HC: 20.1+/-6.90 (NS)	No	No	Intake not among micronutrients assessed	Medium
Geerling [17]	1999	62	P		X	Yes, mean of 50 CD remission reported<br><br>(defined by Van Hees <150)	Prevalence of deficiency not reported<br><br>Median (IQR) in CD remission:<br>19.9 (17.1-23.7) µmol/l<br>Vs<br>HC: 18.0 (15.1-25.2) µmol/l<br>*P* 0.14	Not reported	No	Intake of assessed with FFQ but only comparison between CD active and CD remission *NS*<br><br>Serum Cu in CDa vs CDr higher:<br>26.2 (21.3-38.8) vs 19.9(17.1-23.7) *P* 0.0009	Medium

**Copper** Author [main text ref number]	Year	N CD (HC)	P/R	Data on % Deficiency in CD	CD vs HC	Separate reporting of remission? method used or % remission in group	Prevalence of Deficiency In CD remission group or if not separately reported in CD group Difference vs HC	Supports Copper deficiency being present in CD remission	Supports Copper status being altered in Cd remission in comparison with healthy controls (HC)	Exploration of cause Subgroup analysis or Special tests	Overall Quality (see table 2)
Geerling [18]	2000	23	P		X	Remission not reported separately (CCh): 87% in remission (CDAI <150) Mean (+/- SD) CDAI 96.9+/-66.5	Prevalence of deficiency not reported<br>Mean +/- SD in CD: 23.6+/-8.9 µmol/L<br>Vs HC: 22.2+/7.4µmol/L<br>*NS*	Not reported	No	Intake not among micronutrients assessed	High
MacMaster [20]	2021	59	P	X		All in clinical remission<br><br>53% in biochem remission (BCR) (FCP < 250, CRP <10 Alb >35) (not report separately)	5%<br><10µmol/L men<br><11µmol/L women	Yes	Not reported	Relationship between Alb, FCP and crp explored with Spearman's RHO. Only CRP related (rho=0.41 p<0.001)<br><br>No exploration of phenotypic predictors of deficiency done. Survival analysis vs flare in subsequent 12 months found no relationship	Medium

Manganese

**Manganese** Author [main text ref number]	Year	N CD (HC)	P/R	Data on % Deficiency in CD	CD vs HC	Separate reporting of remission? method used or % remission in group	Prevalence of Deficiency In CD remission group or if not separately reported in CD group<br><br>Difference vs HC	Supports Manganese deficiency being present in CD remission	Supports Manganese status being altered in Cd remission in comparison with healthy controls (HC)	Exploration of cause Subgroup analysis or Special tests	Overall Quality (see table 2)
MacMaster [20]	2021	59	P	X		All in clinical remission<br><br>53% in biochem remission (BCR) (FCP < 250, CRP <10 Alb >35) (not report separately)	Nil reported <70nmol/L	Yes	Not clear / reported	Relationship between Alb, FCP and crp explored with Spearman's RHO. No correlation.<br>No exploration of phenotypic predictors of deficiency done<br><br>Survival analysis vs flare in subsequent 12 months found no relationship	Medium

Magnesium

**Magnesium** Author [main text ref number]	Year	N CD (HC)	P/R	Data on % Deficiency in CD	CD vs HC	Separate reporting of remission? method used or % remission in group	Prevalence of Deficiency In CD remission group or if not separately reported in CD group Difference vs HC	Supports Magnesium deficiency being present in CD remission	Supports Magnesium status being altered in Cd remission in comparison with healthy controls (HC)	Exploration of cause Subgroup analysis or Special tests	Overall Quality (see table 2)
Geerling [16]	1998	32	P	X	X	No separate remission group<br><br>(CCh): Median CDAI 139, 53% remission (CDAI <150)	50% deficient (<0.75mmol/L)<br><br>Mean +/-SD<br>CD: 0.79 +/-0.07 mmol/l<br>Vs<br>HC: 0.85+/-0.07 (*P*<0.01)	Yes	Yes	Assessment of intake did not include Magnesium	Medium
Geerling [18]	2000	23	P		X	Remission not reported separately<br><br>CDAI<br>Mean CDAI 96.9+/-66.5.<br>83% CDAI <150	Prevalence of deficiency not reported<br><br>Mean +/- SD in CD: 0.79+/-0.09 mmol/L<br>Vs<br>HC: 0.82+/0.06mmol/L<br>*NS*	Not clear / reported	No	Assessment of intake did not include Magnesium	Medium

**Magnesium** Author [main text ref number]	Year	N CD (HC)	P/R	Data on % Deficiency in CD	CD vs HC	Separate reporting of remission? method used or % remission in group	Prevalence of Deficiency In CD remission group or if not separately reported in CD group Difference vs HC	Supports Magnesium deficiency being present in CD remission	Supports Magnesium status being altered in Cd remission in comparison with healthy controls (HC)	Exploration of cause Subgroup analysis or Special tests	Overall Quality (see table 2)
Tajika [30]	2004	33 (15)		X	X	No separate reporting of remission. CCh: Mean CDAI 84.1 +/-44.2 (range, 12.6–182.7)	No reported prevalence<br><br>Mean (SD) Mg CD: 2.2+/-0.2 vs HC 2.4+/-0.2 +/-0.3 *P* <0.01	Not reported	Yes	nil	High
Filippi [19]	2006	54	P	X	X	All in remission (CDAI <150) All in remission<br><br>CDAI All subjects needed 3/12 (CDAI <150) Mean CRP 6.6-/-0.8mg/dl (~63nmol/L)	2% (no reference ranges) Estimated from graph only<br><br>Biochemical analysis of HC not reported (either as prevalence of deficiency or results)	Low prevalence only	Not clear / reported	Mean Mg Intake assessed with 3-day food diary in CD and compared vs HC in m and f separately<br><br>Intake in CD f less than HC f 198.29+/-8.33mg/day vs 239.25+/-18.08 *P*<0.01 CD m (n=26) vs HC m (n=9) *NS*	Low
Valentini [35]	2008	94	P	X	X	All in remission (CDAI <150)	28.7% (<0.75nmol/L)<br><br>Conc vs sex matched HC *NS*	Yes	No	nil	High

**Magnesium** Author [main text ref number]	Year	N CD (HC)	P/R	Data on % Deficienc y in CD	CD vs HC	Separate reporting of remission? method used or % remission in group	Prevalence of Deficiency In CD remission group or if not separately reported in CD group Difference vs HC	Supports Magnesium deficiency being present in CD remission	Supports Magnesium status being altered in Cd remission in comparison with healthy controls (HC)	Exploration of cause Subgroup analysis or Special tests	Overall Quality (see table 2)
De Castro [53]	2019	31	P	X		Yes. Remission defined with endoscopy (<5, no deep ulcers or) or MRI with none or creeping fat, deep ulcers or oedema.<br><br>Mean (SD) CDAI of this group 41.35(+/- 41.2)	15.38% (cut off not provided)	Yes	Not reported	Mean Mg compared in remission group to those with endoscopy / MRI activity- *NS*	Medium
MacMaster [20]	2021	59	P	X		All in clinical remission<br><br>53% in biochem remission (BCR) (FCP < 250, CRP <10 alb >35) (not report separately)	7% (0.7mmol/L)	Yes	Not clear / reported	Relationship between alb, FCP and crp explored with Spearman's RHO. Only FCP correlated with serum Mg (rho = -0.26, *p*= 0.044)<br><br>No exploration of phenotypic predictors of deficiency done<br><br>Survival analysis vs flare in subsequent 12 months found no relationship	Medium

Phosphorous

**Phosphorous** Author [main text ref number]	Year	N CD (HC)	P/R	Data on % Deficiency in CD	CD vs HC	Separate reporting of remission? method used or % remission in group	Prevalence of Deficiency In CD remission group or if not separately reported in CD group Difference vs HC	Supports Phosphorous deficiency being present in CD remission	Supports Phosphorous status being altered in Cd remission in comparison with healthy controls (HC)	Exploration of cause Subgroup analysis or Special tests	Overall Quality (see table 2)
Tajika [30]	2004	33 (15)		X	X	No separate reporting of remission. CCh: Mean CDAI 84.1 +/-44.2 (range, 12.6–182.7)	No reported prevalence<br><br>Mean (SD) P CD: 3.2+/-0.6 vs HC 3.5+/-0.6 *P* <0.05	Not reported	Yes		High
Filippi [19]	2006	54	P	X	X	All in remission (CDAI <150) All in remission<br><br>CDAI All subjects needed 3/12 (CDAI <150)<br><br>Mean CRP 6.6-/-0.8mg/dl (~63nmol/L	8% (no reference ranges) Estimated from graph only<br><br>Biochemical analysis of HC not reported (either as prevalence of deficiency or results)	Yes	Not clear / reported	Mean intake assessed with 3-day food diary in CD and compared vs HC in male and female separately<br><br>Intake in CD f (n=28) vs HC f (n=16) and CD m (n=26) vs HC m (n=9)<br>NS<br>All groups > French RDA.	Low

Selenium

**Selenium** Author [main text ref number]	Year	N CD (HC)	P/R	Data on % Deficiency in CD	CD vs HC	Separate reporting of remission? method used or % remission in group	Prevalence of Deficiency In CD remission group or if not separately reported in CD group Difference vs HC	Supports Selenium deficiency being present in CD remission	Supports Selenium status being altered in Cd remission in comparison with healthy controls (HC)	Exploration of cause Subgroup analysis or Special tests	Overall Quality (see table 2)
Geerling [16]	1998	32	P	X	X	No separate remission group<br><br>(CCh): Median CDAI 139, 53% remission (CDAI <150)	50% deficient (<0.91µmol/L):<br><br>Mean +/-SD CD: 0.86 +/-0.14 mmol/l Vs HC: 1.03+/-0.15 (*P*<0.01)	Yes	Yes	Intake not among micronutrients assessed	Medium
Geerling [17]	1999	62	P		X	Yes, mean of 50 CD remission reported<br><br>(defined by Van Hees <150)	Prevalence of deficiency not reported<br><br>Median (IQR) in CD remission: 0.87 (0.76-1.02) µmol/l Vs HC: 1.00 (0.89-1.12) µmol/l *P* <0.0001	Not reported	Yes	Serum Selenium in CDa vs CDr *NS*	Medium

**Selenium** Author [main text ref number]	Year	N CD (HC)	P/R	Data on % Deficiency in CD	CD vs HC	Separate reporting of remission? method used or % remission in group	Prevalence of Deficiency In CD remission group or if not separately reported in CD group Difference vs HC	Supports Selenium deficiency being present in CD remission	Supports Selenium status being altered in Cd remission in comparison with healthy controls (HC)	Exploration of cause Subgroup analysis or Special tests	Overall Quality (see table 2)
Geerling [18]	2000	23	P		X	Remission not reported separately (CCh): 87% in remission (CDAI <150) Mean (+/-SD) CDAI 96.9+/-66.5	Prevalence of deficiency not reported<br><br>Mean +/- SD in CD: 0.79+/-0.09 mmol/L Vs HC: 0.82+/0.06mmol/L *NS*	Not reported	No	Intake not among micronutrients assessed	Medium
Wendland [28]	2000	34	P		X	Not for comparison of Se with HC CCh: 70% in remission (CDAI <150) CDAI Mean +/- SEM 141+/-18.66	No CD diff v HC 0.81 +/-0.0035 µmol/L vs 0.80 +/- 0.036 µmol/L *NS*	Not reported	No	Selenium intake not assessed on 7-day food diary.<br>Se concentration not correlated to CDAI, serum osromucoid or lipid peroxidation as measured by breath pentanes	Medium
Valentini [35]	2008	94	P	X	X	All in remission (CDAI <150)	61.3% (<0.59µmol/L)<br><br>Conc lower in men than sex matched HC. Women- *NS*	Yes	Yes	nil	High

**Selenium** Author [main text ref number]	Year	N CD (HC)	P/R	Data on % Deficiency in CD	CD vs HC	Separate reporting of remission? method used or % remission in group	Prevalence of Deficiency In CD remission group or if not separately reported in CD group Difference vs HC	Supports Selenium deficiency being present in CD remission	Supports Selenium status being altered in Cd remission in comparison with healthy controls (HC)	Exploration of cause Subgroup analysis or Special tests	Overall Quality (see table 2)
MacMaster [20]	2021	59	P	graph		All in clinical remission (CDAI<150)<br><br>53% in biochem remission (BCR) (FCP < 250, CRP <10 Alb >35) (not report separately)	5.1% <0.75µmol/L<br><br>0% (Using RBC Selenium <30.0nmol/g Hb)	Yes	Not reported	Relationship between Alb, FCP and crp explored with Spearman's RHO. Alb and CRP significantly correlated (rho = 0.28; p = 0.007 and rho= -0.28 p = 0.006<br><br>No exploration of phenotypic predictors of deficiency done<br><br>Survival analysis vs flare in subsequent 12 months found no relationship	Medium

Zinc

**Zinc** Author [main text ref number]	Year	N CD (HC)	P/R	Data on % Deficiency in CD	CD vs HC	Separate reporting of remission? method used or % remission in group	Prevalence of Deficiency In CD remission group or if not separately reported in CD group Difference vs HC	Supports Zinc deficiency being present in CD remission	Supports Zinc status being altered in Cd remission in comparison with healthy controls (HC)	Exploration of cause Subgroup analysis or Special tests	Overall Quality (see table 2)
Schoelmerich [21]	1985	38 (19)	P		X	Yes, reports "mildly active" CDAI <150, mild laboratory changes no active symptoms and "inactive" with CDAI<150, no laboratory changes or active symptoms	35% in "mildly active" group 12.5% in "inactive" group (<0.7µg/dl) Zn lower in "inactive" and "mildly active" CD group vs HC 79.1+/-14.3 µg/dl and 86.5+/-13.6 vs 95.3+/-13.0 (p<0.01)	Not reported	Yes	Strong negative correlation with CDAI *R* -0.6226 *p* <0.001	Medium
Geerling [16]	1998	32	P	X	X	No separate remission group<br><br>(CCh): Median CDAI 139, 53% remission (CDAI <150)	50% (<10µmol/L):<br><br>Mean +/-SD CD: 12.0 +/-1.67 µmol/l Vs HC: 13.4+/-2.22 (*P*<0.01)	Yes	Yes	Intake not among micronutrients assessed	Medium

**Zinc** Author [main text ref number]	Year	N CD (HC)	P/R	Data on % Deficiency in CD	CD vs HC	Separate reporting of remission? method used or % remission in group	Prevalence of Deficiency In CD remission group or if not separately reported in CD group Difference vs HC	Supports Zinc deficiency being present in CD remission	Supports Zinc status being altered in Cd remission in comparison with healthy controls (HC)	Exploration of cause Subgroup analysis or Special tests	Overall Quality (see table 2)
Geerling [17]	1999	62	P		X	Yes, mean of 50 CD remission reported<br><br>(defined by Van Hees <150)	Median (IQR) in CD remission: 12.4 (10.8-13.5) µmol/l<br>Vs<br>HC: 13.1 (12.2-13.9) µmol/l<br>*P* 0.007	Not reported	Yes	Intake of assessed with FFQ but only comparison between CD active and CD remission *NS*<br><br>Serum Zinc in CDa vs CDr *NS*	Medium
Geerling [18]	2000	23	P		X	Remission not reported separately (CCh): 87% in remission (CDAI <150) Mean (+/-SD) CDAI 96.9+/-66.5	Mean +/- SD in CD: 12.3+/-3.0 µmol/L<br>Vs<br>HC: 12.9+/1.3µmol/L<br>*NS*	Not reported	No	Intake not among micronutrients assessed	Medium

**Zinc** Author [main text ref number]	Year	N CD (HC)	P/R	Data on % Deficiency in CD	CD vs HC	Separate reporting of remission? method used or % remission in group	Prevalence of Deficiency In CD remission group or if not separately reported in CD group Difference vs HC	Supports Zinc deficiency being present in CD remission	Supports Zinc status being altered in Cd remission in comparison with healthy controls (HC)	Exploration of cause Subgroup analysis or Special tests	Overall Quality (see table 2)
Fillipi [19]	2006	54	P	X	X	All in remission (CDAI <150) All in remission<br><br>CDAI All subjects needed 3/12 (CDAI <150)<br><br>Mean CRP 6.6-/-0.8mg/dl (~63nmol/L	65% (no reference ranges) Estimated from graph only<br><br>Biochemical analysis of HC not reported (either as prevalence of deficiency or results)	Yes	Not clear / reported	Mean Zn intake assessed with 3-day food diary in CD and compared vs HC in male and female separately<br><br>Intake in CD f (n=28) > HC f (n=16)2.42+/-0.37mg vs 0.85+/-0.31mg<br>*P* <0.01<br>CD m (n=26) vs HC m (n=9) NS	Low
Vagianos [34]	2007	84		X		No separate remission CD group<br><br>CCh: 61% in remission (HBI<5)	20.5% (<10μmol/L)	Yes	Not clear / reported	Intake assessed with FFQ then 4-day food diary inc supplements then compared vs adequacy (defined as <66% dietary reference value (DRV)) Zinc intake inadequate in 8.5% CD<br><br>Spearman Correlation of Zinc Intake / 100kcal vs serum No relationship	Medium

**Zinc**<br>Author<br>[main text ref number]	Year	N<br>CD<br>(HC)	P/R	Data on % Deficiency in CD	CD vs HC	Separate reporting of remission? method used or<br>% remission in group	Prevalence of Deficiency<br>In CD remission group or if not separately reported in CD group<br>Difference vs HC	Supports Zinc deficiency being present in CD remission	Supports Zinc status being altered in Cd remission in comparison with healthy controls (HC)	Exploration of cause<br>Subgroup analysis or<br>Special tests	Overall Quality (see table 2)
Valentini<br>[35]	2008	94	P	X	X	All in remission<br>(CDAI <150)	4.3%<br>(<10.3μmol/L)<br><br>Conc vs sex matched HC *NS*	Yes	No	nil	Medium
De Castro<br>[53]	2019	31	P	X		Remission defined with endoscopy (<5, no deep ulcers or) or MRI with none or creeping fat, deep ulcers or oedema.<br>Mean (SD) CDAI of this group 41.35(+/-41.2)	45%<br>(<80mcg/dl)	Yes	Not clear / reported	Mean Zn compared in remission group to those with endoscopy / MRI activity- *NS*	Medium

**Zinc**<br>Author<br>[main text ref number]	Year	N<br>CD<br>(HC)	P/R	Data on % Deficiency in CD	CD vs HC	Separate reporting of remission? method used or<br>% remission in group	Prevalence of Deficiency<br>In CD remission group or if not separately reported in CD group<br>Difference vs HC	Supports Zinc deficiency being present in CD remission	Supports Zinc status being altered in Cd remission in comparison with healthy controls (HC)	Exploration of cause<br>Subgroup analysis or<br>Special tests	Overall Quality (see table 2)
MacMaster<br>[20]	2021	59	P	X		All in clinical remission (CDAI<150)<br><br>53% in biochem remission (BCR) (FCP < 250, CRP <10 Alb >35)<br>(not report separately)	14%<br><br><11.0µmol/L men<br><10µmol/L women	Yes	Not reported	Relationship between Alb, FCP and crp explored with Spearman's RHO. Alb significantly correlated (rho = 0.28; *p* = 0.007)<br><br>Exploration of phenotypic predictors of deficiency found Zn deficiency to be more common in Young age (*p*=0.0.031), low albumin (*p*=0.018), low BMI (*p*<0.001)<br><br>Survival analysis vs flare in subsequent 12 months found Zn deficiency predictive of a shorter time to relapse (HR: 6.9; 95% CI 1.9 to26; p =0.001)<br>This relationship remained after adjustment for CRP and FCP (HR 9.5: 954% CI 2.2 to 42)	Medium
